# Leveraging Genetic Instrumental Variables and Sequencing Analysis to Identify a Prognostic Signature Based on Epithelial Cell Markers in Lung Adenocarcinoma

**DOI:** 10.1111/1759-7714.70244

**Published:** 2026-01-07

**Authors:** Jiaye Lao, Ziqing Han, Xinjing Lou, Jinxuan Ye, Chen Gao, Linyu Wu

**Affiliations:** ^1^ The First Affiliated Hospital of Zhejiang Chinese Medical University (Zhejiang Provincial Hospital of Chinese Medicine) Hangzhou China; ^2^ The First School of Clinical Medicine, Zhejiang Chinese Medical University Hangzhou China

**Keywords:** bulk RNA sequencing, lung adenocarcinoma, Mendelian randomization, prognostic signature, single‐cell RNA sequencing

## Abstract

**Main Problem:**

The treatment and prognosis of lung adenocarcinoma (LUAD) remain challenging. The study aimed to identify prognostic genes and construct a prognostic model for LUAD.

**Methods:**

After identifying malignant alveolar type II (AT2) cells using InferCNV, we applied CytoTRACE, pseudo‐time analysis, Mendelian randomization (MR), and univariate Cox regression analysis to identify prognostic genes. A prognostic model was then developed using an optimized subset of these genes, selected through the least absolute shrinkage and selection operator (LASSO) algorithm. Further analyses included Gene Ontology enrichment analysis and the construction of a protein–protein interaction (PPI) network.

**Results:**

Pseudo‐time analysis identified 3526 dynamically expressed genes during malignant AT2 cell dedifferentiation. Subsequent multi‐omics integration refined the gene selection, yielding four prognostic genes for the final predictive model. The resulting model achieved area under the receiver operating characteristic (ROC) curve (AUC) values of 0.649, 0.675, and 0.654 for predicting 1, 2, and 3‐year overall survival (OS) in the training set, respectively, and was successfully validated in two external cohorts at the corresponding time points. Moreover, survival analysis demonstrated that patients in the high‐risk group had significantly poorer OS than those in the low‐risk group, both in the training set and the validation sets (*p* < 0.01).

**Conclusions:**

The study developed a novel signature based on genes dynamically expressed during malignant AT2 cell dedifferentiation, capable of predicting the prognosis of LUAD patients, and offered four accurate prognostic biomarkers (*ADM*, *MARK4*, *PARVA*, and *RPS6KA1*).

## Graphical abstract

It begins with the identification of malignant AT2 cells, progresses through pseudo‐time analysis and Mendelian randomization to identify prognostic genes, and leads into the construction and validation of the prognostic signature. The visual story also includes functional exploration through Gene Ontology enrichment analysis and protein–protein interaction network analysis.

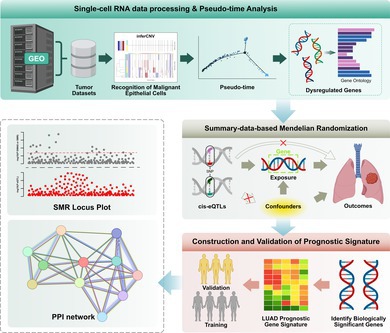

**Abbreviations**
1KGP: the 1000 Genomes Project
AHR: aryl hydrocarbon receptor
AT1: alveolar type I
AT2: alveolar type II
AUC: area under the receiver operating characteristic curve
CNVs: copy number variations
DAPS: Dedifferentiation‐Associated Prognostic Signature
DEGs: differentially expressed genes
DTCNV: dynamic transition copy number variation
DTSL: dynamic transition stemness level
FTO: fat mass and obesity‐associated protein
GEO: gene expression omnibus
GO: Gene Ontology
GWAS: genome‐wide association study
HARPs: hyperacute response proteins
HCNV: high copy number variation
HEIDI: heterogeneity of dependent instruments
HSL: high stemness level
KNN: K‐nearest neighbors
LASSO: the least absolute shrinkage and selection operator
LCNV: low copy number variation
LSL: low stemness level
LUAD: lung adenocarcinoma
MAF: minor allele frequency
MR: Mendelian randomization
OS: overall survival
PCA: principal component analysis
PCs: principal components
PPI: protein–protein interaction
ROC: receiver operating characteristic
scRNA‐seq: single‐cell RNA sequencing
SMR: summary‐data‐based Mendelian randomization
SNP: single‐nucleotide polymorphism

## 1 Introduction

In 2022, lung cancer had the highest global incidence among all malignancies and was the leading cause of cancer‐related deaths [1]. Among all lung cancer types, lung adenocarcinoma (LUAD) has the highest prevalence, accounting for nearly 39% of all lung cancer cases [2]. Although remarkable advances have been made in LUAD therapeutic interventions, the 5‐year overall survival (OS) remains low [3]. Therefore, creating prognostic models to support clinical diagnosis and treatment is crucial.

Bulk RNA sequencing can provide comprehensive gene expression information and is often used to measure gene expression patterns and develop prognostic models [4]. However, it reflects the average expression capacity of genes within cell populations, making cell heterogeneity indistinguishable. Single‐cell RNA sequencing (scRNA‐seq), an advanced sequencing method, enables the analysis of individual cell populations at a single‐cell resolution [5]. This approach facilitates the identification of differentially expressed genes (DEGs) involved in tumor dedifferentiation [6, 7]. Although many LUAD prognostic models based on marker genes of specific cell types have been developed [8, 9, 10], their predictive power still needs improvement. A primary reason for this limitation is the inherently small number of individuals in scRNA‐seq studies, which diminishes the statistical strength of the identified markers and further hampers model performance [11, 12].

Mendelian randomization (MR), a genetic epidemiology technique, is used to infer causal relationships between genetic variants, exposures, and disease outcomes [13]. A major benefit of MR is its dependence on extensive genetic data, offering enough statistical strength for accurate gene screening [14]. The large sample size ensures accurate identification of genes with a true causal relationship to LUAD, thereby mitigating the propensity for false positives observed in other scRNA‐seq studies with a limited number of individuals [11, 15]. Despite the key role of MR, its combination with multi‐omics is still relatively rare.

By integrating multi‐omics approaches that combine the resolution of single‐cell sequencing with the statistical robustness of MR, we aimed to establish a single‐cell‐level risk prognostic model for LUAD that can predict patient prognosis.

## 2 Methods

### 2.1 Data Collection

#### 2.1.1 Single‐Cell RNA Sequencing Datasets

A total of 43 LUAD samples with scRNA‐seq data were obtained from the Gene Expression Omnibus (GEO) database. The datasets included GSE131907 (*n* = 11), GSE153935 (*n* = 12), and GSE119911 (*n* = 20).

#### 2.1.2 Bulk RNA Datasets

To expand the scope of the study, we downloaded a LUAD RNA‐seq dataset (GSE68465) from the GEO database, which contained 442 LUAD samples. These samples, including complete survival time and status information, enabled the construction of biomarker‐based risk prognostic models for LUAD. Additionally, two additional LUAD RNA‐seq datasets (GSE50081 and GSE42127) containing complete survival time and status information were downloaded to verify the robustness of the novel risk prognostic model in the independent datasets.

#### 2.1.3 Summary‐Data‐Based Mendelian Randomization (SMR) Datasets

##### 2.1.3.1 Exposure Data

We obtained statistically significant *cis*‐expression quantitative trait loci (*cis*‐eQTLs) (*p*‐value < 5 × 10^−8^, within ±1000 kb of each probe, false discovery rate < 0.05) from the eQTLGen Consortium [16, 17]. We imputed the single‐nucleotide polymorphisms (SNPs) in the eQTL dataset using genotyped data aligned to the 1000 Genomes Project (1KGP) reference panels [18]. Only SNPs with a minor allele frequency (MAF) > 0.01 were included in the analyses.

##### 2.1.3.2 Outcome Data

GWAS summary data on LUAD were retrieved from the MRC‐IEU (11 245 cases and 54 619 controls, European ancestry, HG19/GRCh37; ieu‐a‐984).

### 2.2 Single‐Cell RNA Data Processing

To ensure the quality of scRNA‐seq data, we filtered out cells expressing fewer than 200 genes or more than 7000 genes, genes expressed in fewer than three single cells, and cells with more than 5% mitochondrial genes.

Following normalization, the gene expression matrices were transformed into a Seurat object. The “Harmony” function was used to eliminate batch effects.

For dimensionality reduction, principal component analysis (PCA) was applied to the integration‐transformed expression matrix using the top 2000 highly variable genes. JackStraw analysis was implemented to identify optimal principal components (PCs), and the top 50 PCs were chosen for cell clustering. Then, we determined the nearest neighbors of each cell using Euclidean distance in PCA, followed by constructing a K‐nearest neighbors (KNN) map. The resolution parameter for clustering was set to 1.5.

Cell lineages were assigned based on the expression of canonical marker genes, such as *KRT5* and *KRT14* for basal cells, *CAV1* and *CAV2* for alveolar epithelial type I (AT1) cells, and *SFTPB* and *SFTPC* for AT2 cells [19].

### 2.3 Recognition of Malignant Epithelial Cells

To distinguish malignant cells, our analysis employed InferCNV, a widely adopted tool for inferring copy number variations (CNVs) [20]. The T cell was used as a reference in InferCNV to determine whether other cells exhibit substantial chromosomal CNV [21].

### 2.4 Refine the Starting Point of the Inferred Trajectories Using CytoTRACE

To further refine the starting point of the inferred trajectories, we employed the CytoTRACE algorithm, a computational method that predicts cellular differentiation states from scRNA‐seq data [22]. This analysis assigned a CytoTRACE score to each epithelial cell, which reflects its differentiation state. CytoTRACE scores range from 0 to 1, with higher scores indicating greater stemness.

### 2.5 Identification of DEGs by Pseudo‐Time Analysis

We performed a pseudo‐time analysis on malignant AT2 cells to explore differences at the genome level in the progression of LUAD using the “Monocle” package [23]. First, using the “newCellDataSet” function, we constructed a CellDataSet object. To choose order genes for trajectory construction, we used the highly variable genes identified by the “FindVariableFeatures” function and “differentialGeneTest” function. The “DDRTree” algorithm was employed for dimensionality reduction. Then, we ordered the cells in pseudotime using the “orderCells” function. To identify genes whose expression significantly changes along the developmental trajectory, we performed differential expression analysis using the “differentialGeneTest” function with the model formula “~sm.ns(Pseudotime).” Genes with Benjamini‐Hochberg‐adjusted *q*‐values < 1 × 10^−10^ were considered statistically significant as pseudotime‐dependent DEGs.

### 2.6 The Key Genes Associated With LUAD Were Screened by SMR

SMR analysis was performed using SMR software version 1.3.1, developed by the Yang Laboratory [24]. Pleiotropy in SMR occurs when a single SNP is associated with multiple genes, as assessed using the heterogeneity in dependent instruments (HEIDI) test. We performed the HEIDI test in the SMR software [25]. The HEIDI value below 0.05 suggested pleiotropy [26]. Genes exhibiting pleiotropy were excluded from further analysis.

### 2.7 Construction and Validation of Prognostic Signature Based on Key Genes

Using univariate Cox regression analysis, we evaluated the prognosis of each gene dynamically expressed during malignant AT2 cell dedifferentiation that was shown by SMR to be causally associated with LUAD. Genes significantly associated with the OS were identified as key genes (*p* < 0.05). To minimize overfitting, the LASSO proportional hazards regression was used to determine core prognostic genes. Finally, the risk prognostic model was established as the weighted sum of messenger RNA expression levels for genes identified by multivariate Cox regression analysis.

We calculated optimal cutoff values for the risk score in the GEO cohort using X‐tile software from Yale University and divided the scores into high‐ and low‐score groups. To validate the prognostic power of Dedifferentiation‐Associated Prognostic Signature (DAPS), the area under the ROC curve (AUC) was calculated [27]. The Kaplan–Meier method was used for survival analysis. The robustness of this signature was verified in two independent GEO datasets (GSE50081 and GSE42127). The “sva” package removed batch effects between internal and external datasets.

### 2.8 Gene Ontology (GO) Enrichment Analysis and Protein–Protein Interaction (PPI) Network

GO enrichment analysis was implemented using the “clusterProfiler” package, and GO annotations were based on “org.Hs.eg.db.” Statistical significance for enrichment was set at *p* < 0.05 [28, 29]. The PPI network of prognostic genes was constructed using the STRING database (https://cn.string‐db.org/).

## 3 Results

### 3.1 Identification of Epithelial Cell Marker Gene Expression Profiles

**FIGURE 1:**
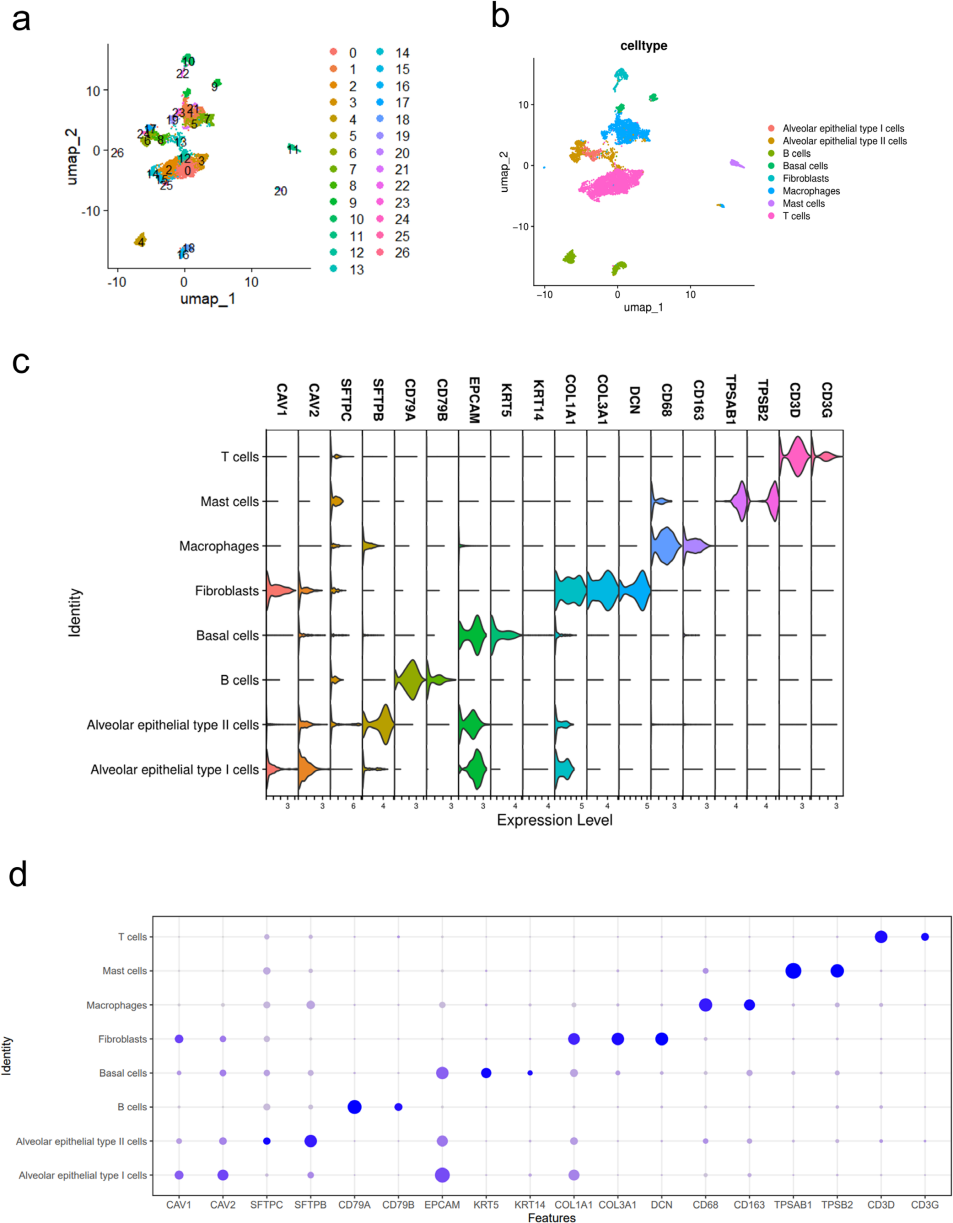
ScRNA data processing. (a) t‐SNE plot depicting 27 distinct cell clusters. (b) The cell types identified by marker genes. (c) The violin plot showing the expression levels of canonical marker genes across different cell identities. (d) The dot plot showing the expression levels of canonical marker genes across different cell identities.

With LUAD scRNA‐seq data of GSE119911 (*n* = 3493), GSE131907 (*n* = 5000), and GSE153935 (*n* = 5155), we obtained transcriptomic matrices of 13 648 cells derived from 43 LUAD patients (Figure 1a). Based on canonical marker genes, major cell populations were identified, including T cells, macrophages, B cells, AT1 cells, AT2 cells, basal cells, fibroblasts, and mast cells (Figure 1b). The expression of different canonical marker genes showed significant differences between various groups, indicating that the grouping was reliable (Figure 1c,d).

**FIGURE 2:**
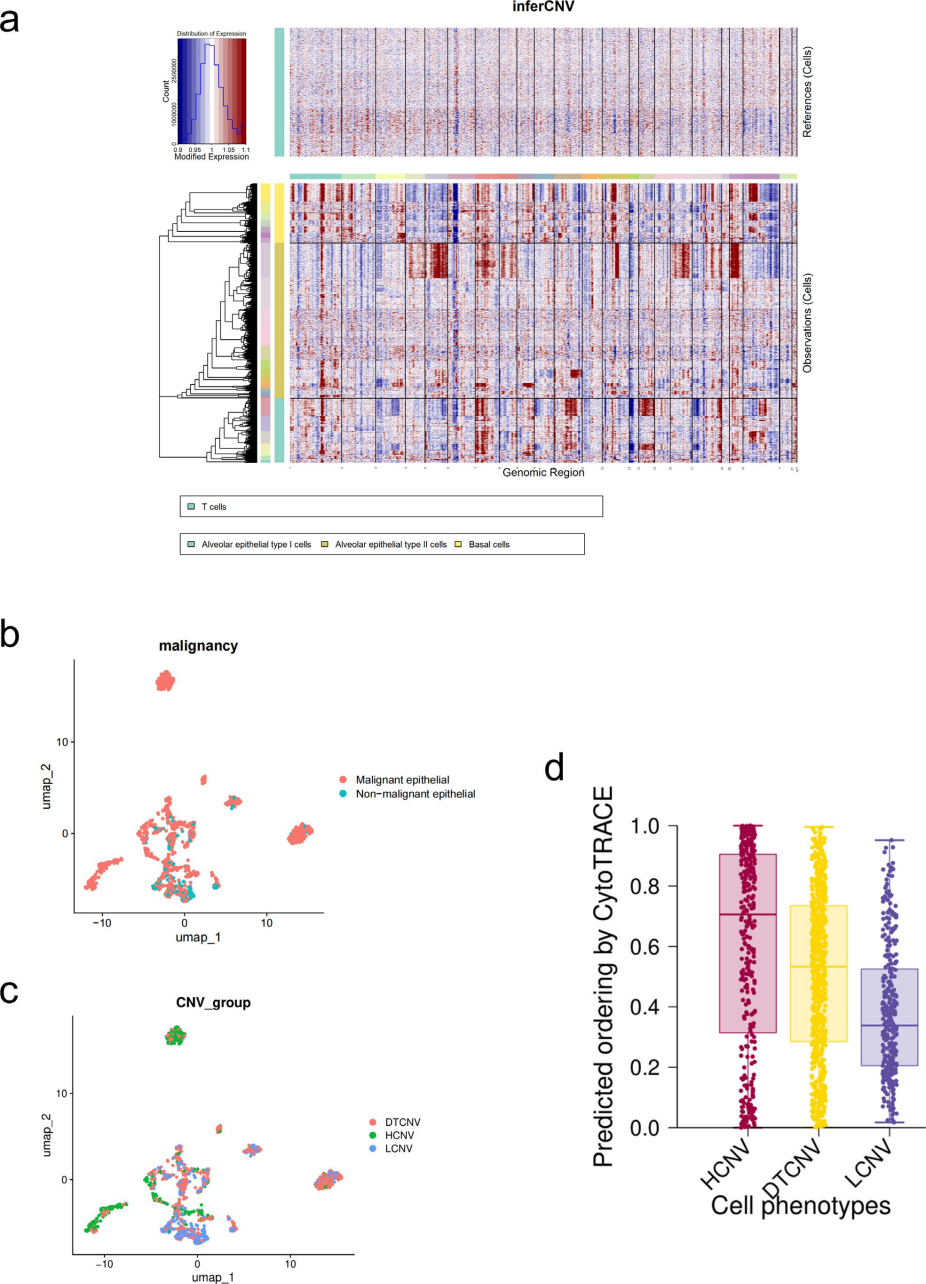
CNV‐based classification. (a) CNV heatmap of LUAD epithelial cells. (b) UMAP visualization of epithelial cells reveals malignant and non‐malignant populations by CNV profiles. (c) This classification system was used to quantify each tumor cell's deviation from the copy number levels of normal cells, with the states designated as low (LCNV; ≤ 25th percentile), dynamic transition (DTCNV; 25th–75th percentile), and high (HCNV; ≥ 75th percentile). (d) Boxplots comparing CytoTRACE scores among CNV groups.

To identify malignant cells, we inferred CNVs in three epithelial cell types, including AT1, AT2, and basal cells, using T cells as a reference (Figure 2a). We identified clusters of cells showing high levels of inferred copy number aberrations and classified them as malignant cells (Figure 2b).

To assess the reliability of CNV scores, we used a quartile‐based segmentation method to categorize tumor cells according to their CNV scores, defining three distinct CNV states (LCNV, DTCNV, and HCNV) (Figure 2c). The results showed that HCNV cells had higher CytoTRACE scores (Kruskal–Wallis test, *p*‐value = 0.000664), indicating a greater degree of dedifferentiation (Figure 2d). This aligns with our expectation that as tumor cells evolve, they undergo ongoing dedifferentiation, along with a rising frequency of CNVs. This correlation further supports the validity of the CNV scoring system.

AT2 cells are recognized as the primary stem cell population in the distal lung epithelium and the most common cells of origin for LUAD [30]. Their dysregulation is a key event in LUAD pathogenesis. Meanwhile, the genes expressed by AT1 cells and AT2 cells are distinctly different. Therefore, we believe that focusing on changes in AT2 cells can exclude irrelevant signals, thereby enabling a more focused effort to elucidate the biological mechanisms underlying lung cancer development.

**FIGURE 3:**
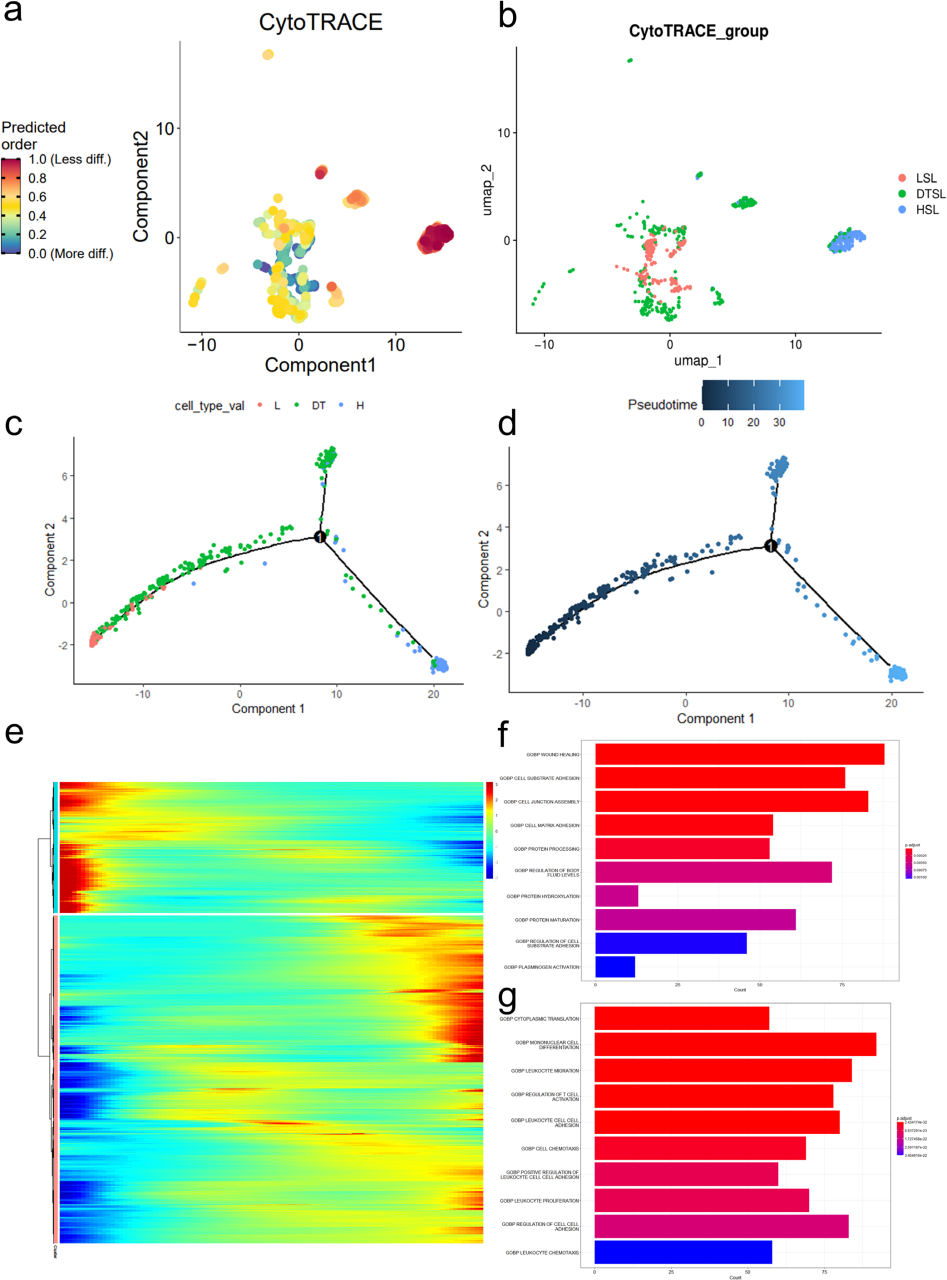
Pseudo‐time analysis of CytoTRACE‐based Groups and Gene Ontology (GO) analysis. (a) The CytoTRACE scores of malignant AT2 cells. (b) UMAP of epithelial cells stratified into LSL, DTSL, and HSL groups. (c) The trajectory plot maps cells colored by cell type. (d) The trajectory plot maps cells colored by pseudo‐time. (e) Heatmap of differentially expressed genes along pseudo‐time. (f) GO enrichment analysis of upregulated genes along pseudo‐time. (g) GO enrichment analysis of downregulated genes along pseudo‐time.

To determine the starting point of the trajectory, we used the CytoTRACE algorithm to recalculate the CytoTRACE score for each malignant AT2 cell before the pseudo‐time analysis (Figure 3a). We noted notable differences in the extent of dedifferentiation among these cells. To explore how these dedifferentiation variations impact LUAD prognosis, we segmented malignant AT2 cells into three stemness levels based on quartiles: low (LSL; ≤ 25th percentile), dynamic transition (DTSL; 25th–75th percentile), and high (HSL; ≥ 75th percentile) (Figure 3b). Pseudo‐time trajectory analysis showed that cell states gradually transitioned from LSL to HSL via DTSL (Figure 3c,d). This further confirms a relatively continuous degree of dedifferentiation among cells within the tumor. Some genes show increased expression during this process, while others are downregulated (Figure 3e). Notably, the expression levels of *ADM*, *MARK4*, *PARVA*, and *RPS6KA1* change along the developmental trajectory (Figure S1). To comprehensively characterize these transcriptional dynamics, we used the “differentialGeneTest” function and identified 3526 genes with statistically significant expression patterns along the pseudo‐time trajectory (*q*‐value < 1 × 10^−10^). These genes were selected for further analysis (Tables S1 and S2).

To further investigate these genes showing differential expression along the pseudo‐time trajectory, we performed a GO enrichment analysis. Genes upregulated along this trajectory were significantly enriched in processes essential for tissue remodeling and invasion, such as wound healing, cell–matrix adhesion, plasminogen activation, and protein maturation (Figure 3f). These processes are vital for tumor cell proliferation and invasion. In contrast, genes downregulated in LUAD were significantly enriched in immune‐related biological processes, such as leukocyte migration, regulation of T cell activation, leukocyte cell–cell adhesion, and leukocyte chemotaxis (Figure 3g). The suppression of these processes indicates an immunosuppressive tumor microenvironment.

### 3.2 The Key Genes Associated With LUAD Were Screened by SMR

SMR was used for further screening. Genes from the IEU database with *p*_SMR values greater than 0.01 or the HEIDI values below 0.05 were excluded. This filtering process preserved 226 genes (Table S3). After intersecting these 226 genes with the 3526 genes identified through single‐cell sequencing, 24 genes were retained (Table 1).

**TABLE 1:** Summary‐data‐based Mendelian randomization results for 24 intersection genes.

Gene	b_SMR	se_SMR	*p*_SMR	*p*_HEIDI
ADM	−0.129	0.049	0.008	0.655
AQP3	0.321	0.097	< 0.001	0.167
ARID3B	−0.367	0.099	< 0.001	0.393
ARL1	0.264	0.100	0.008	0.662
CD40	−0.111	0.036	0.002	0.582
CD5	−0.215	0.083	0.010	0.189
FUCA1	0.094	0.036	0.010	0.649
GMFG	0.353	0.092	< 0.001	0.122
GPR161	0.499	0.188	0.008	0.287
HLA‐DPA1	0.066	0.025	0.009	0.369
HLA‐G	−0.097	0.031	0.002	0.102
IER3	0.187	0.047	< 0.001	0.059
LIMA1	0.147	0.049	0.003	0.401
MARK4	−0.137	0.045	0.002	0.498
MPZL2	−0.152	0.024	< 0.001	0.136
PARVA	−0.455	0.127	< 0.001	0.063
PSMA6	−0.384	0.149	0.010	0.557
RPS6KA1	−0.243	0.082	0.003	0.540
SELL	0.083	0.028	0.003	0.957
STAG3	−0.105	0.039	0.007	0.418
TUBB6	0.073	0.027	0.007	0.449
UCKL1	−0.231	0.060	< 0.001	0.207
MBTPS1	0.114	0.036	0.002	0.682
RNASET2	0.070	0.018	< 0.001	0.812

Abbreviations: b_SMR, the beta value of the SMR; se_SMR, the standard deviation value of the SMR.

### 3.3 Establishment of the Four‐Gene Prognostic Signature Based on Key Genes

**FIGURE 4:**
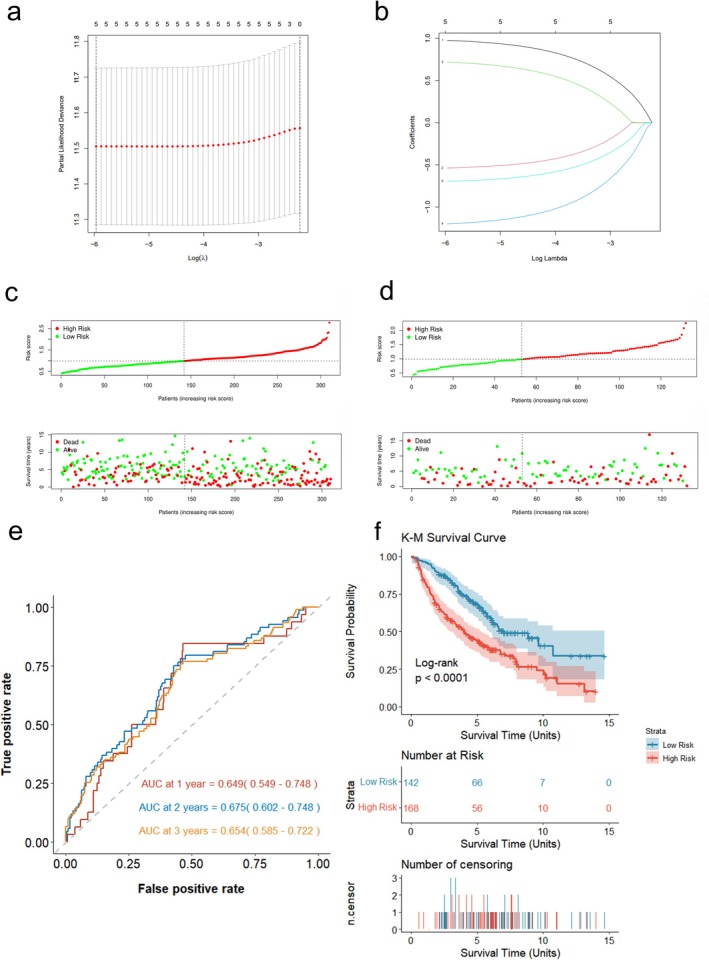
Establishment of the DAPS in the GEO LUAD cohort. (a) LASSO cross‐validation for lambda (left dashed line marks lambda‐min, the value at which the deviance was minimized and which was selected as the optimal penalty parameter). (b) LASSO coefficient paths. (c) The distribution of risk scores and survival status of the training group. (d) The distribution of risk scores and survival status of the internal validation group. (e) ROC curves of the DAPS for predicting the risk of death at 1, 2, and 3 years in the train group. (f) Kaplan–Meier curves of the DAPS in the train group.

To construct a risk prognostic model based on these 24 intersection genes, the GSE68465 cohort was used to perform a univariate Cox regression analysis. Five genes (*ADM*, *HLA‐DPA1*, *MARK4*, *PARVA*, and *RPS6KA1*) were identified as significantly associated with OS and were selected for further modeling (Table 2). LASSO Cox regression analysis was then applied to these five genes to prevent overfitting (Figure 4a,b).

**TABLE 2:** Five key genes significantly related to OS.

Gene	HR	HR.95L	HR.95H	*p*
ADM	3.209	1.243	8.286	0.016
HLA‐DPA1	0.344	0.126	0.937	0.037
MARK4	2.727	1.005	7.401	0.049
PARVA	0.285	0.106	0.764	0.013
RPS6KA1	0.289	0.106	0.787	0.015

Abbreviations: HR, hazard ratio; HR.95H, hazard ratio 95% confidence interval upper bound; HR.95L, hazard ratio 95% confidence interval lower bound.

Finally, stepwise multivariate Cox regression analysis was used to optimize the risk prognostic model by selecting the four optimal prognostic genes:

$$\begin{array}{c} \text{risk score}=\left(1.009581684\times \mathrm{ADM}\;\text{expression}\right)\\ \;+\left(0.757128322\times \text{MARK4}\;\text{expression}\right)\\ \;+\left(-1.304656383\times \text{PARVA}\;\text{expression}\right)\\ \;+\left(-0.888578312\times \mathrm{RPS}6\text{KA1}\;\text{expression}\right). \end{array}$$

The optimal risk score cutoff of 0.985 was first determined via X‐tile exclusively on the training cohort (GSE68465, *n* = 310), classifying it into high‐risk (*n* = 168) and low‐risk (*n* = 142) groups. For the internal validation cohort (*n* = 132), we uniformly applied this 0.985 cutoff, resulting in 79 high‐risk and 53 low‐risk patients. Figure 4c,d exhibited the distribution of survival status and risk scores, indicating that there was longer survival time in the low‐risk group. To better visualize the predictive ability of the risk model, AUC for OS was calculated. And the 1‐, 2‐, and 3‐year AUC values were 0.649, 0.675, 0.654, respectively, in the training group (Figure 4e). Kaplan–Meier curves of the DAPS also suggested higher death risks in the high‐risk group (*p* < 0.0001) (Figure 4f).

**FIGURE 5:**
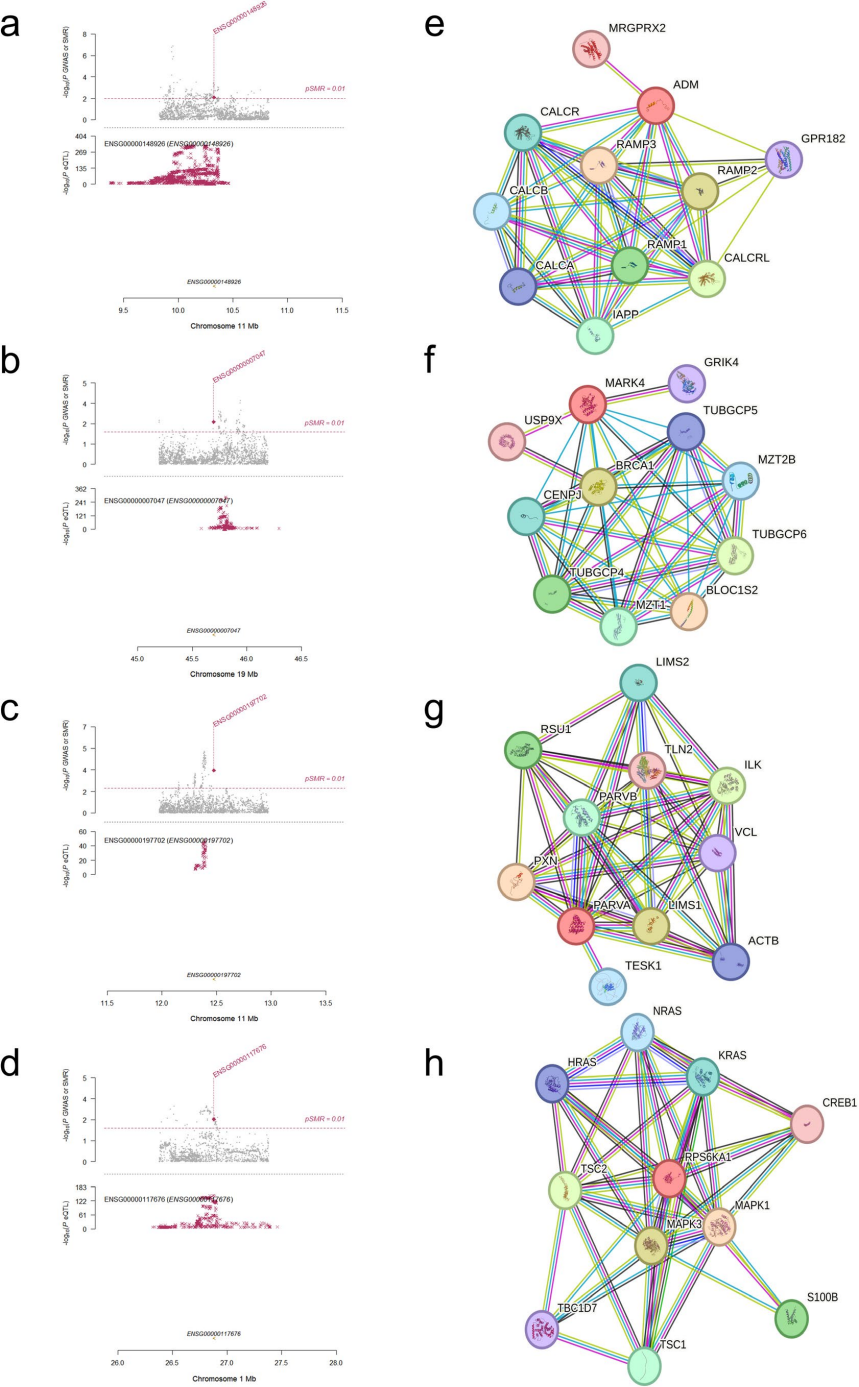
SMR locus plots, and PPI networks of the four prognostic genes. (a–d) SMR locus plots of *ADM, MARK4, PARVA*, and *RPS6KA1*. The genes that survived after the SMR and HEIDI tests were highlighted in red. (e–h) The PPI networks of *ADM, MARK4, PARVA*, and *RPS6KA1*.

To show the causal relationship between these four prognostic genes and LUAD, we drew locus plots to present the MR results (Figure 5a–d). All four prognostic genes easily passed the test, suggesting a potential causal relationship between them and LUAD.

Then, using the STRING database, the PPI network of prognostic genes was constructed (Figure 5e–h). The 40 genes most closely associated with these four prognostic genes are listed.

### 3.4 External Validation of the Robustness of the Cox Model in Independent Cohorts

**FIGURE 6:**
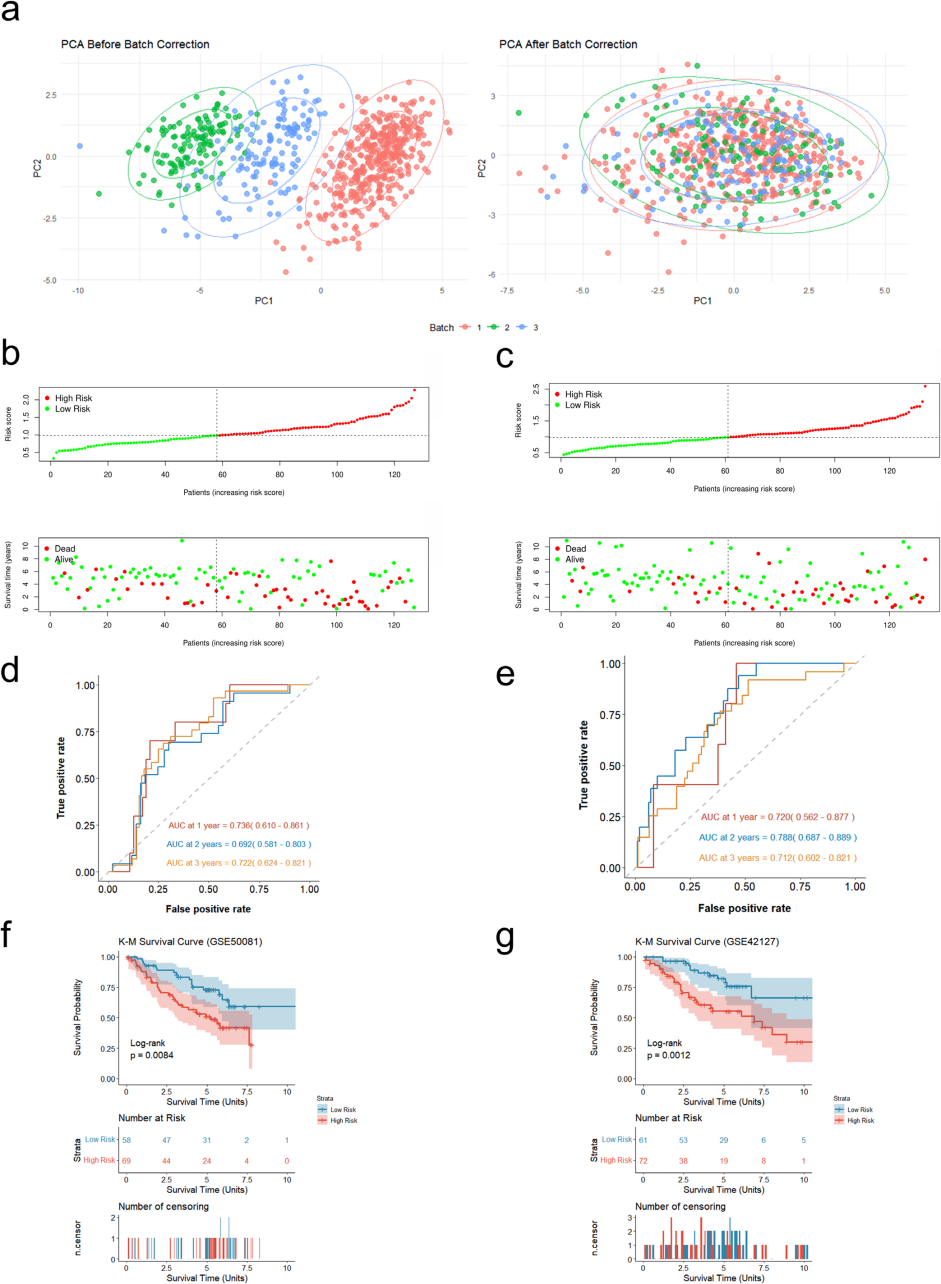
External validation of the robustness of the cox model in independent cohorts. (a) PCA visualization of batch effect correction. Data1 is the train set (GSE68465). Data2 (GSE50081) and data3 (GSE42127) are the external data sets. (b) The distribution of risk scores and survival status in GSE50081. (c) The distribution of risk scores and survival status in GSE42127. (d) ROC curves of the DAPS for predicting the risk of death at 1‐, 2‐, and 3‐years in GSE50081. (e) ROC curves of the DAPS for predicting the risk of death at 1‐, 2‐, and 3‐years in GSE42127. (f) Kaplan–Meier curves of the DAPS in GSE50081. (g) Kaplan–Meier curves of the DAPS in GSE42127.

We included two external GEO cohorts (GSE50081 and GSE42127) to validate the robustness of the DAPS. After removing batch effects between internal and external datasets, the same forecast formula was used to calculate the score of each sample in the two external GEO cohorts (Figure 6a). Using the training‐set‐derived optimal cutoff of 0.985, patients in GSE50081 were categorized into low‐risk (*n* = 58) and high‐risk (*n* = 69) groups, while those in GSE42127 were categorized into low‐risk (*n* = 61) and high‐risk (*n* = 72) groups.

Figure 6b,c showed that there were more deaths in the high‐risk group in both external GEO cohorts. The 1‐, 2‐, and 3‐year AUC values were 0.736, 0.692, 0.722 for GSE50081, and the 1‐, 2‐, and 3‐year AUC values were 0.720, 0.788, 0.712 for GSE42127, respectively (Figure 6d,e). Kaplan–Meier analysis demonstrated that the low‐risk group had a superior prognosis compared with the high‐risk group (*p* < 0.01), indicating that this Cox model was a reliable prognostic instrument for LUAD (Figure 6f,g).

## 4 Discussion

To develop a clinically useful prognostic model for LUAD, we combined scRNA‐seq with InferCNV, CytoTRACE, and SMR analyses to identify 24 genes that are dynamically expressed during malignant AT2 cell dedifferentiation and are likely causal for LUAD. From these candidate genes, we developed a four‐gene risk prognostic signature for LUAD. Kaplan–Meier survival analysis further confirmed that patients in the low‐risk group had a significantly better prognosis compared to those in the high‐risk group (*p* < 0.01), confirming the reliability of this Cox model as a prognostic tool for LUAD. The 2‐year AUC of the DAPS was 0.788 in the independent validation set, demonstrating superior predictive power compared to existing prognostic models [8, 9, 10].

Our pseudo‐time analysis effectively mapped the dedifferentiation pathway in AT2 cells, showing a coordinated increase in genes related to wound healing and cell–matrix adhesion, while genes involved in antitumor responses were generally downregulated. Specifically, as Harold F Dvorak compared cancer to “a wound that does not heal,” tumor cells often hijack the wound healing pathway, and the hyperactivation of this pathway creates the key conditions for cancer invasion [31]. Similarly, the abnormal increase in genes related to cell–matrix adhesion suggests that tumor cells are actively interacting with the extracellular matrix through molecules like integrins. These cells are dynamically adjusting their adhesive abilities to promote stromal invasion, gain access to blood vessels, and spread metastatically [32]. Meanwhile, the downregulation of genes controlling key immune processes such as leukocyte migration, T cell activation, and chemotaxis establishes an immunosuppressive tumor microenvironment that undermines immune surveillance and promotes tumor invasion and metastasis [33].

The DAPS was composed of four key genes (*ADM*, *MARK4*, *PARVA*, and *RPS6KA1*), which are correlated with the prognosis of LUAD patients. *ADM* encodes adrenomedullin, a multifunctional peptide that acts as a direct transcriptional target of the cigarette smoke‐induced aryl hydrocarbon receptor (AHR) pathway [34, 35, 36]. It plays a key role in increasing tumor aggressiveness by creating an immunosuppressive microenvironment, closely linked to immune checkpoint molecules like PD‐L1, which promotes T‐cell exhaustion and aids in immune evasion [35, 37]. Elevated *ADM* expression is consistently recognized as a powerful negative prognostic biomarker, significantly associated with shorter OS in LUAD patients [37]. Meanwhile, *MARK4*, a serine/threonine kinase, enhances tumor cell stress resistance by phosphorylating the Fat Mass and Obesity‐Associated Protein (FTO) under stress conditions, which subsequently promotes the hyperacute translation of Hyperacute Response Proteins (HARPs) within *γ*‐tubulin‐associated translation microdomains [38, 39]. Due to its central role in LUAD progression, *MARK4* is considered a promising therapeutic target, and its upstream inhibitor, microRNA‐515‐5p, has been shown to be associated with prolonged patient survival [40, 41].

Our approach to a more extensive multi‐omics strategy, particularly one that incorporates MR, is a strong initiative. As noted by Jordan W. Squair, scRNA‐seq studies are often prone to false positives, necessitating robust statistical validation [11, 12]. It serves as a model that future researchers should consider when developing new prognostic models, not only for LUAD but also for other cancers.

While the findings are promising, this study has several limitations. Firstly, while our model performed robustly in validation cohorts of diverse origins, the pseudo‐time analysis was confined to AT2 cells, potentially limiting the generalizability to non‐AT2‐derived LUAD subtypes. Secondly, it depended on a public dataset, and confirming the predictive ability of the identified signature will require a large‐scale prospective clinical study. Lastly, the mechanistic analyses conducted in this study were descriptive; no in vitro or in vivo experiments were performed to further investigate the underlying biological molecular mechanisms.

In conclusion, we have identified and validated a four‐gene prognostic risk model based on genes dynamically expressed during malignant AT2 cell dedifferentiation, demonstrating strong potential to predict prognosis in LUAD patients. This signature could serve as a valuable tool in clinical decision‐making.

## Author Contributions

**Jiaye Lao** and **Ziqing Han:** conceptualization, investigation, formal analysis, data curation, visualization, writing – original draft, writing – reviewing and editing. **Xinjing Lou:** visualization, writing – reviewing and editing. **Jinxuan Ye:** writing – original draft, writing – reviewing and editing. **Chen Gao** and **Linyu Wu:** conceptualization, methodology, resources, writing – reviewing and editing, supervision, project administration. All authors read and approved the final manuscript.

## Funding

This research was supported by Medical and Health Science and Technology Project of Zhejiang Province (Grant No. 2024KY129, 2025KY093, 2024KY132), Jinhua Public Welfare Technology Application Research Project (Grant No. 2024‐4‐121), Zhejiang Province Traditional Chinese Medicine Science and Technology Plan Project (Grant No. 2025ZL302, 2025ZS012), and the Research Project of Zhejiang Chinese Medical University (Grant No. 2022FSYYZY08). The study sponsors had no role in the study design, in the collection, analysis, and interpretation of data; in the writing of the manuscript; and in the decision to submit the manuscript for publication.

## Ethics Statement

The authors have nothing to report.

## Consent

The authors have nothing to report.

## Conflicts of Interest

The authors declare no conflicts of interest.

## Supporting information


**Figure S1:** tca70244‐sup‐0001‐FigureS1.tiff.

**Table S1:** Genes downregulated along the pseudotime trajectory (*n* = 1005).

**Table S2:** Genes upregulated along the pseudotime trajectory (*n* = 2521).

**Table S3:** Genes from the IEU database with *p*_SMR values greater than 0.01 and HEIDI values less than 0.01 (*n* = 226).

## Data Availability

The data that support the findings of this study are available in Gene Expression Omnibus, eQTLGen Consortium, and MRC‐IEU at https://www.ncbi.nlm.nih.gov/geo/, https://opengwas.io/datasets/, and https://opengwas.io/datasets/. These data were derived from the following resources available in the public domain: GSE131907, https://www.ncbi.nlm.nih.gov/geo/query/acc.cgi?acc=GSE131907; GSE153935, https://www.ncbi.nlm.nih.gov/geo/query/acc.cgi?acc=GSE153935; GSE119911, https://www.ncbi.nlm.nih.gov/geo/query/acc.cgi?acc=GSE119911; GSE68465, https://www.ncbi.nlm.nih.gov/geo/query/acc.cgi?acc=GSE68465; GSE50081, https://www.ncbi.nlm.nih.gov/geo/query/acc.cgi?acc=GSE50081; GSE42127, https://www.ncbi.nlm.nih.gov/geo/query/acc.cgi?acc=GSE42127; *cis*‐eQTLs data, https://eqtlgen.org/cis‐eqtls.html; ieu‐a‐984, https://opengwas.io/datasets/ieu‐a‐984.
