# Supplementary figures and images for: Leveraging Genetic Instrumental Variables and Sequencing Analysis to Identify a Prognostic Signature Based on Epithelial Cell Markers in Lung Adenocarcinoma
Source: Thorac Cancer. 2026 Jan 7;17(1):e70244. doi: 10.1111/1759-7714.70244 (PMC12779403; doi:10.1111/1759-7714.70244)

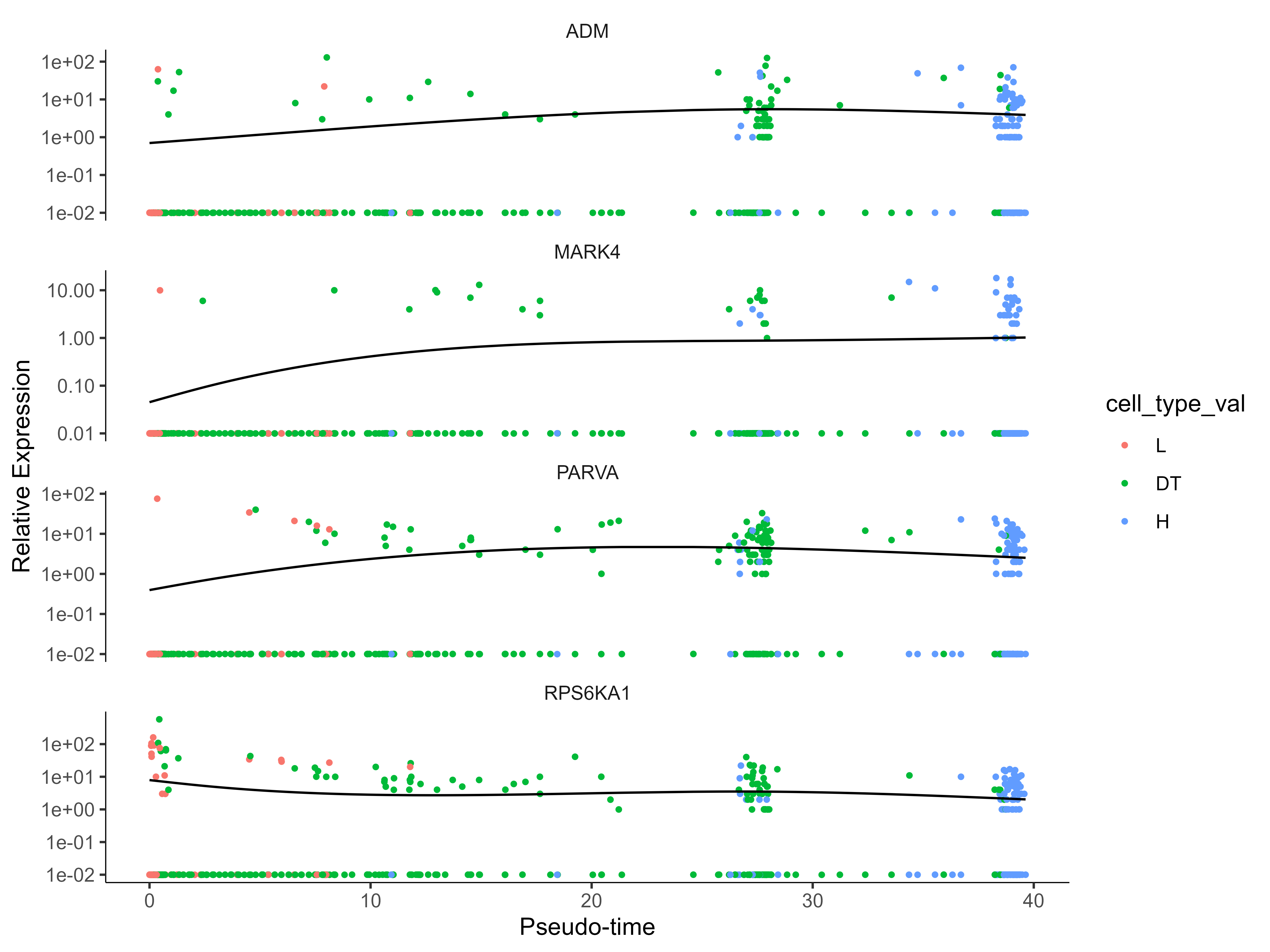

Supplement: Supplementary file 1 — Figure S1: tca70244‐sup‐0001‐FigureS1.tiff. [file TCA-17-e70244-s004.tiff]
