# Supplementary material for: Leveraging Genetic Instrumental Variables and Sequencing Analysis to Identify a Prognostic Signature Based on Epithelial Cell Markers in Lung Adenocarcinoma
Source: Thorac Cancer. 2026 Jan 7;17(1):e70244. doi: 10.1111/1759-7714.70244 (PMC12779403; doi:10.1111/1759-7714.70244)
Supplement: Supplementary file 2 — Table S1: Genes downregulated along the pseudotime trajectory (n = 1005). [file TCA-17-e70244-s002.docx]

Supplementary Table 1 Genes downregulated along the pseudotime trajectory (n=1005)

| No. | Gene | No. | Gene | No. | Gene | No. | Gene |
| --- | --- | --- | --- | --- | --- | --- | --- |
| 1 | A2M | 253 | DCN | 505 | LDLRAD4 | 757 | RPL27A |
| 2 | ABI3 | 254 | DDHD1 | 506 | LEF1 | 758 | RPL28 |
| 3 | ABTB1 | 255 | DDT | 507 | LGALS2 | 759 | RPL3 |
| 4 | ACADS | 256 | DDX21 | 508 | LIG1 | 760 | RPL30 |
| 5 | ACAP1 | 257 | DDX24 | 509 | LILRB1 | 761 | RPL32 |
| 6 | ACP5 | 258 | DDX39A | 510 | LILRB4 | 762 | RPL35 |
| 7 | ACRBP | 259 | DEF6 | 511 | LIMD2 | 763 | RPL35A |
| 8 | ADA | 260 | DEK | 512 | LINC00341 | 764 | RPL36A |
| 9 | ADAP2 | 261 | DENND1C | 513 | LINC00824 | 765 | RPL5 |
| 10 | ADGRF3 | 262 | DEPDC1 | 514 | LINC00892 | 766 | RPL7A |
| 11 | AGER | 263 | DEPDC1B | 515 | LINC00963 | 767 | RPLP1 |
| 12 | AIF1 | 264 | DIAPH3 | 516 | LINC01094 | 768 | RPLP2 |
| 13 | AKAP5 | 265 | DLEU7-AS1 | 517 | LINC01133 | 769 | RPS10 |
| 14 | AKT3 | 266 | DLGAP5 | 518 | LINC01272 | 770 | RPS11 |
| 15 | ALDH16A1 | 267 | DMC1 | 519 | LIPA | 771 | RPS12 |
| 16 | ALDH1A1 | 268 | DNAJA1 | 520 | LMNB1 | 772 | RPS13 |
| 17 | ALOX5 | 269 | DNAJB4 | 521 | LMO4 | 773 | RPS15 |
| 18 | ALOX5AP | 270 | DNASE1L3 | 522 | LOC100419170 | 774 | RPS15A |
| 19 | ANAPC15 | 271 | DNMT1 | 523 | LOC101927069 | 775 | RPS16 |
| 20 | ANKRD44 | 272 | DOCK2 | 524 | LOC284454 | 776 | RPS17 |
| 21 | ANP32A | 273 | DOK1 | 525 | LPAR5 | 777 | RPS18 |
| 22 | ANP32B | 274 | DOK2 | 526 | LPXN | 778 | RPS19 |
| 23 | AOAH | 275 | DOK3 | 527 | LRMP | 779 | RPS2 |
| 24 | AP1S2 | 276 | DPP7 | 528 | LRRC26 | 780 | RPS20 |
| 25 | APBB1IP | 277 | DUOX1 | 529 | LRRC8C | 781 | RPS24 |
| 26 | APH1A | 278 | DUSP2 | 530 | LSP1 | 782 | RPS25 |
| 27 | APOBEC3G | 279 | DUSP4 | 531 | LST1 | 783 | RPS27 |
| 28 | APOC1 | 280 | DUSP5 | 532 | LTB | 784 | RPS27A |
| 29 | APOE | 281 | DUT | 533 | LUM | 785 | RPS28 |
| 30 | APOLD1 | 282 | E2F2 | 534 | LY86 | 786 | RPS29 |
| 31 | AQP3 | 283 | E2F7 | 535 | LYZ | 787 | RPS3 |
| 32 | ARAP2 | 284 | EEF1B2 | 536 | MAGEA4 | 788 | RPS4X |
| 33 | AREG | 285 | EEF1D | 537 | MAGOH | 789 | RPS5 |
| 34 | ARHGAP11A | 286 | EEF2 | 538 | MAP1A | 790 | RPS6 |
| 35 | ARHGAP15 | 287 | EFHD2 | 539 | MAP2K3 | 791 | RPS6KA1 |
| 36 | ARHGAP25 | 288 | EHD1 | 540 | MAP2K6 | 792 | RPS6KA4 |
| 37 | ARHGAP30 | 289 | EID3 | 541 | MAP3K8 | 793 | RPS7 |
| 38 | ARHGAP4 | 290 | EIF3F | 542 | MAP4K1 | 794 | RPS8 |
| 39 | ARHGAP45 | 291 | EIF3G | 543 | MAPKAP1 | 795 | RPS9 |
| 40 | ARHGAP9 | 292 | EIF3M | 544 | 1-Mar | 796 | RPSA |
| 41 | ARHGDIB | 293 | ELMO1 | 545 | MARCO | 797 | RRM1 |
| 42 | ARHGEF1 | 294 | EMP3 | 546 | MAT2A | 798 | RRM2 |
| 43 | ARHGEF6 | 295 | ENSA | 547 | MATK | 799 | RTF1 |
| 44 | ARID3A | 296 | ERH | 548 | MCL1 | 800 | RTKN2 |
| 45 | ARID3B | 297 | ESCO2 | 549 | MCM10 | 801 | RUNX3 |
| 46 | ARID5A | 298 | ETS1 | 550 | MCOLN2 | 802 | S100A2 |
| 47 | ARPC1B | 299 | EVI2A | 551 | MEF2C | 803 | S100A4 |
| 48 | ARPC2 | 300 | EVI2B | 552 | METAP2 | 804 | S100B |
| 49 | ARPC5 | 301 | EVL | 553 | MFNG | 805 | S1PR4 |
| 50 | ARRB2 | 302 | EZH2 | 554 | MIDN | 806 | SAMHD1 |
| 51 | ASF1B | 303 | FABP4 | 555 | MIF4GD | 807 | SAMSN1 |
| 52 | ASPM | 304 | FAM101B | 556 | MKI67 | 808 | SAPCD2 |
| 53 | ATAD2 | 305 | FAM129A | 557 | MME | 809 | SASH3 |
| 54 | ATF3 | 306 | FAM179A | 558 | MMP12 | 810 | SATB1 |
| 55 | ATG16L1 | 307 | FAM26F | 559 | MMP19 | 811 | SCGB1A1 |
| 56 | ATG7 | 308 | FAM46C | 560 | MMS22L | 812 | SDCBP |
| 57 | ATP2A3 | 309 | FAM49B | 561 | MNDA | 813 | SDHC |
| 58 | ATP5E | 310 | FAM64A | 562 | MOB1A | 814 | SDS |
| 59 | ATP5EP2 | 311 | FAM78A | 563 | MPEG1 | 815 | SELL |
| 60 | ATP5G2 | 312 | FAM83D | 564 | MPP1 | 816 | SELPLG |
| 61 | ATP5L | 313 | FANCD2 | 565 | MRC1 | 817 | SEMA4A |
| 62 | ATP5L2 | 314 | FANCI | 566 | MRPL48 | 818 | SEMA6D |
| 63 | AURKB | 315 | FBXO5 | 567 | MRPS18C | 819 | SEPP1 |
| 64 | AXL | 316 | FCER1A | 568 | MS4A1 | 820 | SEPT6 |
| 65 | BAG1 | 317 | FCER1G | 569 | MS4A14 | 821 | SET |
| 66 | BCL11A | 318 | FCGR1A | 570 | MS4A4A | 822 | SFPQ |
| 67 | BCL11B | 319 | FCGR2A | 571 | MS4A6A | 823 | SFTPA1 |
| 68 | BCL2A1 | 320 | FCGR2B | 572 | MS4A7 | 824 | SFTPC |
| 69 | BGN | 321 | FCGR3A | 573 | MSC | 825 | SFTPD |
| 70 | BHMT2 | 322 | FCHO1 | 574 | MSR1 | 826 | SH2D1A |
| 71 | BID | 323 | FCHSD2 | 575 | MT1G | 827 | SH2D2A |
| 72 | BIN2 | 324 | FCN1 | 576 | MTHFS | 828 | SH3BP1 |
| 73 | BIRC5 | 325 | FDPS | 577 | MTRNR2L1 | 829 | SHCBP1 |
| 74 | BLM | 326 | FEN1 | 578 | MYADM | 830 | SIGLEC9 |
| 75 | BRD1 | 327 | FERMT3 | 579 | MYBL2 | 831 | SIMC1 |
| 76 | BRD2 | 328 | FFAR4 | 580 | MYL9 | 832 | SIRPA |
| 77 | BRD4 | 329 | FGD5 | 581 | MYO1F | 833 | SIRPG |
| 78 | BTG1 | 330 | FGL2 | 582 | MYO9B | 834 | SLA |
| 79 | BTG2 | 331 | FGR | 583 | MZB1 | 835 | SLA2 |
| 80 | BTK | 332 | FKBP8 | 584 | NAP1L1 | 836 | SLAMF1 |
| 81 | BUB1 | 333 | FLJ35934 | 585 | NAPSB | 837 | SLAMF8 |
| 82 | BUD31 | 334 | FLT3 | 586 | NBEAL1 | 838 | SLC25A16 |
| 83 | C10orf54 | 335 | FMNL1 | 587 | NCAPD3 | 839 | SLC2A3 |
| 84 | C11orf96 | 336 | FMNL3 | 588 | NCAPG | 840 | SLC2A6 |
| 85 | C12orf75 | 337 | FMO2 | 589 | NCAPG2 | 841 | SLC31A2 |
| 86 | C18orf54 | 338 | FNBP1 | 590 | NCAPH | 842 | SLC37A2 |
| 87 | C19orf43 | 339 | FNDC5 | 591 | NCF1 | 843 | SLC7A7 |
| 88 | C1orf162 | 340 | FOLR2 | 592 | NCF2 | 844 | SMAP2 |
| 89 | C1orf228 | 341 | FOSB | 593 | NCF4 | 845 | SMC4 |
| 90 | C1orf54 | 342 | FOSL2 | 594 | NCKAP1L | 846 | SMCHD1 |
| 91 | C1QA | 343 | FOXM1 | 595 | NCL | 847 | SMIM17 |
| 92 | C1QB | 344 | FPR1 | 596 | NDRG2 | 848 | SMIM5 |
| 93 | C1QBP | 345 | FPR2 | 597 | NEIL3 | 849 | SNCA |
| 94 | C1QC | 346 | FPR3 | 598 | NFIL3 | 850 | SNHG6 |
| 95 | C21orf58 | 347 | FRMD4B | 599 | NKG7 | 851 | SNRNP70 |
| 96 | C22orf39 | 348 | FTH1 | 600 | NLRC4 | 852 | SNX10 |
| 97 | C5AR1 | 349 | FTL | 601 | NOTCH1 | 853 | SOBP |
| 98 | C7orf61 | 350 | FUOM | 602 | NPL | 854 | SOD3 |
| 99 | C8orf59 | 351 | FUS | 603 | NR4A1 | 855 | SP140 |
| 100 | CA2 | 352 | FXYD5 | 604 | NR4A2 | 856 | SPAG5 |
| 101 | CACNA2D2 | 353 | FYB | 605 | NR4A3 | 857 | SPC24 |
| 102 | CALM3 | 354 | FYN | 606 | NRN1 | 858 | SPC25 |
| 103 | CALML3 | 355 | GABPB1 | 607 | NTM | 859 | SPDL1 |
| 104 | CALML4 | 356 | GABRA2 | 608 | NTRK2 | 860 | SPI1 |
| 105 | CALML5 | 357 | GAS6 | 609 | NTS | 861 | SPIB |
| 106 | CAMK4 | 358 | GATA3 | 610 | NUCKS1 | 862 | SPN |
| 107 | CAPG | 359 | GBP5 | 611 | NUDT1 | 863 | SPNS3 |
| 108 | CAPZA1 | 360 | GIMAP4 | 612 | NUF2 | 864 | SPOCK2 |
| 109 | CAPZB | 361 | GIMAP6 | 613 | NUSAP1 | 865 | SPP1 |
| 110 | CARD8 | 362 | GIMAP7 | 614 | OAZ1 | 866 | SPRR2A |
| 111 | CBFA2T3 | 363 | GIMAP8 | 615 | ODF2 | 867 | SPTY2D1 |
| 112 | CBX6 | 364 | GLIPR2 | 616 | OIP5-AS1 | 868 | SRGN |
| 113 | CCDC51 | 365 | GLTSCR2 | 617 | OLFM4 | 869 | SRSF9 |
| 114 | CCDC85B | 366 | GMFG | 618 | OLR1 | 870 | SSH2 |
| 115 | CCDC88B | 367 | GMIP | 619 | OPRM1 | 871 | STAB1 |
| 116 | CCL13 | 368 | GNG2 | 620 | OSCAR | 872 | STAP1 |
| 117 | CCL18 | 369 | GNLY | 621 | OSM | 873 | STARD8 |
| 118 | CCL2 | 370 | GOLGA7 | 622 | OST4 | 874 | STAT4 |
| 119 | CCL20 | 371 | GPNMB | 623 | P2RX5 | 875 | STK17B |
| 120 | CCL23 | 372 | GPR183 | 624 | P2RY10 | 876 | STK4 |
| 121 | CCL3 | 373 | GPR65 | 625 | PABPC1 | 877 | STMN1 |
| 122 | CCL4 | 374 | GPR84 | 626 | PAFAH1B1 | 878 | SUMF2 |
| 123 | CCL4L2 | 375 | GPRIN3 | 627 | PALD1 | 879 | SUSD3 |
| 124 | CCL5 | 376 | GPSM3 | 628 | PAQR8 | 880 | SYF2 |
| 125 | CCL7 | 377 | GPX2 | 629 | PARVB | 881 | TACC3 |
| 126 | CCL8 | 378 | GRAP2 | 630 | PARVG | 882 | TAF13 |
| 127 | CCNA2 | 379 | GRK2 | 631 | PBK | 883 | TAF1D |
| 128 | CCNB1 | 380 | GRWD1 | 632 | PCNA | 884 | TAGAP |
| 129 | CCNB2 | 381 | GTSE1 | 633 | PDCD4 | 885 | TAGLN |
| 130 | CCND2 | 382 | GYPC | 634 | PDE4B | 886 | TAOK3 |
| 131 | CCNI | 383 | GZMA | 635 | PDE6B | 887 | TARP |
| 132 | CCR1 | 384 | GZMB | 636 | PDIA6 | 888 | TBC1D10C |
| 133 | CCR2 | 385 | GZMH | 637 | PDLIM2 | 889 | TBCC |
| 134 | CCR5 | 386 | GZMK | 638 | PEA15 | 890 | TBCD |
| 135 | CCR6 | 387 | HAMP | 639 | PFDN6 | 891 | TCEANC2 |
| 136 | CCR7 | 388 | HAUS6 | 640 | PFN1 | 892 | TCF4 |
| 137 | CCSAP | 389 | HAUS8 | 641 | PGC | 893 | TDP2 |
| 138 | CD14 | 390 | HAVCR2 | 642 | PHACTR1 | 894 | TFEC |
| 139 | CD163 | 391 | HBEGF | 643 | PHF11 | 895 | TFRC |
| 140 | CD1E | 392 | HCAR3 | 644 | PHF5A | 896 | TGFB1 |
| 141 | CD2 | 393 | HCK | 645 | PHLDA1 | 897 | TGM1 |
| 142 | CD247 | 394 | HCLS1 | 646 | PI3 | 898 | THEMIS |
| 143 | CD27 | 395 | HCST | 647 | PIF1 | 899 | THEMIS2 |
| 144 | CD300A | 396 | HELLS | 648 | PIK3AP1 | 900 | TIGIT |
| 145 | CD300E | 397 | HHEX | 649 | PIK3CD | 901 | TLE4 |
| 146 | CD300LF | 398 | HIST1H1A | 650 | PIK3CG | 902 | TMCC1 |
| 147 | CD36 | 399 | HIST1H1B | 651 | PIK3R5 | 903 | TMEM119 |
| 148 | CD37 | 400 | HIST1H1D | 652 | PILRA | 904 | TMEM156 |
| 149 | CD3D | 401 | HIST1H1E | 653 | PIM1 | 905 | TMEM160 |
| 150 | CD3E | 402 | HIST1H2AG | 654 | PIM2 | 906 | TMEM37 |
| 151 | CD3G | 403 | HIST1H2AH | 655 | PLA2G1B | 907 | TMEM44 |
| 152 | CD4 | 404 | HIST1H2BH | 656 | PLA2G7 | 908 | TMEM52B |
| 153 | CD48 | 405 | HIST1H4C | 657 | PLCB2 | 909 | TMEM88 |
| 154 | CD5 | 406 | HIST1H4F | 658 | PLCL2 | 910 | TMPO |
| 155 | CD52 | 407 | HIST2H2AC | 659 | PLD4 | 911 | TNF |
| 156 | CD53 | 408 | HJURP | 660 | PLEK | 912 | TNFAIP1 |
| 157 | CD6 | 409 | HK3 | 661 | PLEKHA2 | 913 | TNFAIP3 |
| 158 | CD68 | 410 | HLA-DPA1 | 662 | PLEKHO1 | 914 | TNFRSF18 |
| 159 | CD69 | 411 | HLA-DQA2 | 663 | PLEKHO2 | 915 | TNFRSF1B |
| 160 | CD7 | 412 | HLA-DQB1 | 664 | PLIN2 | 916 | TNFRSF25 |
| 161 | CD79A | 413 | HMGB2 | 665 | PLK1 | 917 | TNFRSF4 |
| 162 | CD79B | 414 | HMOX1 | 666 | PLK3 | 918 | TNFSF4 |
| 163 | CD80 | 415 | HNRNPA1 | 667 | PLXDC2 | 919 | TNFSF8 |
| 164 | CD83 | 416 | HNRNPD | 668 | PNRC1 | 920 | TNNT1 |
| 165 | CD84 | 417 | HNRNPU | 669 | POLQ | 921 | TOP2A |
| 166 | CD86 | 418 | HPGDS | 670 | POLR2M | 922 | TOX |
| 167 | CD8A | 419 | HSD17B14 | 671 | POU2F2 | 923 | TPGS1 |
| 168 | CD8B | 420 | HSD3BP4 | 672 | PPDPF | 924 | TPM3 |
| 169 | CD93 | 421 | HSPA1B | 673 | PPIB | 925 | TPSAB1 |
| 170 | CD96 | 422 | HSPA6 | 674 | PPP1CA | 926 | TPSB2 |
| 171 | CDC20 | 423 | HSPD1 | 675 | PPP1R14A | 927 | TPT1 |
| 172 | CDC25C | 424 | ICAM4 | 676 | PPP1R15A | 928 | TPX2 |
| 173 | CDC45 | 425 | ICOS | 677 | PPP1R16B | 929 | TRA2B |
| 174 | CDCA2 | 426 | ID2 | 678 | PPP3R1 | 930 | TRAF3IP3 |
| 175 | CDCA3 | 427 | ID3 | 679 | PRAME | 931 | TRAPPC10 |
| 176 | CDCA5 | 428 | IDI1 | 680 | PRC1 | 932 | TRAT1 |
| 177 | CDCA8 | 429 | IER3 | 681 | PRDM1 | 933 | TREM2 |
| 178 | CDH24 | 430 | IFFO1 | 682 | PRELID1 | 934 | TRIM11 |
| 179 | CDK1 | 431 | IFI30 | 683 | PREX1 | 935 | TROAP |
| 180 | CDK6 | 432 | IFNG | 684 | PRF1 | 936 | TRPV2 |
| 181 | CDKN1B | 433 | IGLL5 | 685 | PRKCB | 937 | TSHZ3 |
| 182 | CDKN2C | 434 | IGSF6 | 686 | PRKCH | 938 | TTC39C |
| 183 | CDKN3 | 435 | IKZF1 | 687 | PRKCQ | 939 | TTK |
| 184 | CEBPZ | 436 | IKZF3 | 688 | PRPF38B | 940 | TTL |
| 185 | CECR1 | 437 | IL10RA | 689 | PRR11 | 941 | TTN |
| 186 | CENPE | 438 | IL16 | 690 | PSMB7 | 942 | TUBA1B |
| 187 | CENPF | 439 | IL18R1 | 691 | PSMG2 | 943 | TUBB |
| 188 | CENPK | 440 | IL1B | 692 | PSTPIP1 | 944 | TUBB6 |
| 189 | CENPM | 441 | IL1RN | 693 | PTCH2 | 945 | TYMP |
| 190 | CEP135 | 442 | IL21R | 694 | PTGDS | 946 | TYROBP |
| 191 | CEP55 | 443 | IL2RA | 695 | PTGER4 | 947 | UAP1 |
| 192 | CFD | 444 | IL2RB | 696 | PTGS2 | 948 | UBA2 |
| 193 | CHAC2 | 445 | IL2RG | 697 | PTMS | 949 | UBA52 |
| 194 | CHAF1A | 446 | IL4I1 | 698 | PTPN7 | 950 | UBE2C |
| 195 | CHCHD10 | 447 | IL7R | 699 | PTPRC | 951 | UBE2E2 |
| 196 | CHIT1 | 448 | INCENP | 700 | PTPRCAP | 952 | UBE2T |
| 197 | CHST11 | 449 | IQGAP2 | 701 | PTPRZ1 | 953 | UCP2 |
| 198 | CHST2 | 450 | IQSEC1 | 702 | PTTG1 | 954 | UQCRB |
| 199 | CIB2 | 451 | IRF4 | 703 | PYHIN1 | 955 | UTY |
| 200 | CITED2 | 452 | IRF7 | 704 | RAB5C | 956 | VASP |
| 201 | CKAP2 | 453 | IRF8 | 705 | RAB8B | 957 | VAV1 |
| 202 | CKAP2L | 454 | ITGA4 | 706 | RAC2 | 958 | VENTX |
| 203 | CKS1B | 455 | ITGAL | 707 | RAD21 | 959 | VRK1 |
| 204 | CLEC10A | 456 | ITGAX | 708 | RAD51 | 960 | VSIG4 |
| 205 | CLEC4A | 457 | ITGB2 | 709 | RAD51AP1 | 961 | VTI1B |
| 206 | CLEC4E | 458 | ITGB3BP | 710 | RALGDS | 962 | WAS |
| 207 | CLECL1 | 459 | ITGB7 | 711 | RANBP1 | 963 | WDPCP |
| 208 | CLSPN | 460 | ITK | 712 | RAP1A | 964 | WDR76 |
| 209 | CMKLR1 | 461 | ITM2A | 713 | RARRES1 | 965 | WIF1 |
| 210 | CNOT6L | 462 | IVNS1ABP | 714 | RASAL3 | 966 | WIPF1 |
| 211 | CNTRL | 463 | JAK3 | 715 | RASGRP2 | 967 | XCL1 |
| 212 | COL10A1 | 464 | JAML | 716 | RASSF2 | 968 | XIST |
| 213 | COL3A1 | 465 | JCHAIN | 717 | RB1 | 969 | YBX1 |
| 214 | COL4A1 | 466 | JUN | 718 | RBBP6 | 970 | YWHAH |
| 215 | COL4A2 | 467 | JUNB | 719 | RBM25 | 971 | YY1 |
| 216 | COL5A2 | 468 | JUND | 720 | RCSD1 | 972 | ZBTB7A |
| 217 | COMMD8 | 469 | KBTBD2 | 721 | RECQL | 973 | ZC3H12D |
| 218 | CORO1A | 470 | KCNAB2 | 722 | REEP1 | 974 | ZC3HAV1 |
| 219 | COTL1 | 471 | KCNMA1 | 723 | RELT | 975 | ZCCHC2 |
| 220 | COX16 | 472 | KCNQ1OT1 | 724 | RETN | 976 | ZEB2 |
| 221 | CPA3 | 473 | KDM1B | 725 | RGCC | 977 | ZFP36L2 |
| 222 | CPT1A | 474 | KDM6B | 726 | RGS1 | 978 | ZNF143 |
| 223 | CPVL | 475 | KIAA0101 | 727 | RGS10 | 979 | ZNF230 |
| 224 | CREM | 476 | KIAA1524 | 728 | RGS16 | 980 | ZNF331 |
| 225 | CRTAM | 477 | KIAA1549L | 729 | RGS18 | 981 | ZNF394 |
| 226 | CSF1R | 478 | KIAA1551 | 730 | RGS19 | 982 | ZNF445 |
| 227 | CSF2RB | 479 | KIF11 | 731 | RGS2 | 983 | ZNF506 |
| 228 | CSF3 | 480 | KIF14 | 732 | RHOG | 984 | ZNF556 |
| 229 | CSF3R | 481 | KIF15 | 733 | RHOH | 985 | ZNF585B |
| 230 | CSRNP1 | 482 | KIF20B | 734 | RILPL2 | 986 | ZNF683 |
| 231 | CST7 | 483 | KIF23 | 735 | RLF | 987 | ZNF90 |
| 232 | CSTA | 484 | KIF2C | 736 | RMRP | 988 | ZWINT |
| 233 | CTLA4 | 485 | KIFC1 | 737 | RNASE6 | 989 | GNB2L1 |
| 234 | CTSS | 486 | KLF2 | 738 | RNASET2 | 990 | H3F3A |
| 235 | CTSW | 487 | KLF6 | 739 | RND1 | 991 | IGKC |
| 236 | CTSZ | 488 | KNL1 | 740 | RNF125 | 992 | IL8 |
| 237 | CXCL13 | 489 | KNSTRN | 741 | RNF166 | 993 | MT-ATP6 |
| 238 | CXCL16 | 490 | KPNA2 | 742 | RNF38 | 994 | MT-CO1 |
| 239 | CXCL3 | 491 | KRT5 | 743 | RNVU1-19 | 995 | MT-CO2 |
| 240 | CXCL8 | 492 | LAG3 | 744 | RPH3A | 996 | MT-CO3 |
| 241 | CXCL9 | 493 | LAIR1 | 745 | RPL10 | 997 | MT-CYB |
| 242 | CXCR3 | 494 | LAMP5 | 746 | RPL10A | 998 | MT-ND1 |
| 243 | CXCR4 | 495 | LAPTM5 | 747 | RPL11 | 999 | MT-ND2 |
| 244 | CXCR6 | 496 | LAT | 748 | RPL12 | 1000 | MT-ND3 |
| 245 | CXorf21 | 497 | LAT2 | 749 | RPL13A | 1001 | MT-ND4 |
| 246 | CYBA | 498 | LAYN | 750 | RPL14 | 1002 | MT-ND5 |
| 247 | CYBB | 499 | LBR | 751 | RPL18A | 1003 | MT-RNR1 |
| 248 | CYTH1 | 500 | LBX2 | 752 | RPL19 | 1004 | MT-RNR2 |
| 249 | CYTH4 | 501 | LCK | 753 | RPL21 | 1005 | SLC25A6 |
| 250 | CYTIP | 502 | LCP1 | 754 | RPL22 |  |  |
| 251 | DAB2 | 503 | LCP2 | 755 | RPL23A |  |  |
| 252 | DBF4 | 504 | LDLR | 756 | RPL26 |  |  |
