# Supplementary material for: Leveraging Genetic Instrumental Variables and Sequencing Analysis to Identify a Prognostic Signature Based on Epithelial Cell Markers in Lung Adenocarcinoma
Source: Thorac Cancer. 2026 Jan 7;17(1):e70244. doi: 10.1111/1759-7714.70244 (PMC12779403; doi:10.1111/1759-7714.70244)
Supplement: Supplementary file 3 — Table S2: Genes upregulated along the pseudotime trajectory (n = 2521). [file TCA-17-e70244-s003.docx]

Supplementary Table 2 Genes upregulated along the pseudotime trajectory (n=2521)

| No. | gene | No. | gene | No. | gene | No. | gene |
| --- | --- | --- | --- | --- | --- | --- | --- |
| 1 | AAMDC | 632 | ELOVL7 | 1263 | LOC339803 | 1894 | RWDD3 |
| 2 | AAR2 | 633 | EML5 | 1264 | LOC375196 | 1895 | S100A10 |
| 3 | AARS | 634 | EMP1 | 1265 | LOC389641 | 1896 | S100A13 |
| 4 | AARS2 | 635 | ENC1 | 1266 | LOC389906 | 1897 | S100A16 |
| 5 | AASS | 636 | ENDOD1 | 1267 | LOC401320 | 1898 | S100A8 |
| 6 | ABCA12 | 637 | ENG | 1268 | LOC403323 | 1899 | S100A9 |
| 7 | ABCA17P | 638 | ENGASE | 1269 | LOC440300 | 1900 | S100P |
| 8 | ABCA4 | 639 | ENKD1 | 1270 | LOC440934 | 1901 | SAA2 |
| 9 | ABCB6 | 640 | ENOPH1 | 1271 | LOC441242 | 1902 | SAMD12 |
| 10 | ABCC1 | 641 | ENOSF1 | 1272 | LOC553103 | 1903 | SAMD15 |
| 11 | ABCC3 | 642 | ENPP3 | 1273 | LOC554206 | 1904 | SAMD9L |
| 12 | ABCC6 | 643 | ENTPD6 | 1274 | LOC554223 | 1905 | SAP30L |
| 13 | ABCC6P2 | 644 | EPB41L1 | 1275 | LOC642852 | 1906 | SAP30L-AS1 |
| 14 | ABCF2 | 645 | EPB41L4A | 1276 | LOC643733 | 1907 | SAR1B |
| 15 | ABHD11 | 646 | EPCAM | 1277 | LOC644656 | 1908 | SARAF |
| 16 | ABHD12 | 647 | EPDR1 | 1278 | LOC648987 | 1909 | SARS2 |
| 17 | ABHD16A | 648 | EPHB2 | 1279 | LOC652276 | 1910 | SBDSP1 |
| 18 | ABHD17C | 649 | EPHB4 | 1280 | LOC654342 | 1911 | SCAMP1 |
| 19 | ABHD4 | 650 | EPHX3 | 1281 | LOC727896 | 1912 | SCAND1 |
| 20 | ABLIM3 | 651 | EPHX4 | 1282 | LOC728024 | 1913 | SCAND2P |
| 21 | ACACA | 652 | ERBB2 | 1283 | LOC728554 | 1914 | SCAP |
| 22 | ACCS | 653 | ERBB3 | 1284 | LOC728730 | 1915 | SCARB2 |
| 23 | ACLY | 654 | ERBIN | 1285 | LOC728743 | 1916 | SCIN |
| 24 | ACO1 | 655 | ERCC4 | 1286 | LOC729348 | 1917 | SCML1 |
| 25 | ACOT7 | 656 | ERCC6 | 1287 | LOC729966 | 1918 | SCN9A |
| 26 | ACOX2 | 657 | ERGIC1 | 1288 | LOC730098 | 1919 | SCNN1A |
| 27 | ACSF2 | 658 | ERGIC3 | 1289 | LOC730102 | 1920 | SCNN1B |
| 28 | ACSF3 | 659 | ERI2 | 1290 | LOC90784 | 1921 | SCOC |
| 29 | ACSL1 | 660 | ERO1A | 1291 | LONP2 | 1922 | SCRN1 |
| 30 | ACSL5 | 661 | ERO1B | 1292 | LOX | 1923 | SCRN2 |
| 31 | ACSM1 | 662 | ERVMER34-1 | 1293 | LPCAT1 | 1924 | SDC1 |
| 32 | ACTN1 | 663 | ESRP1 | 1294 | LPGAT1 | 1925 | SDC3 |
| 33 | ACVR1 | 664 | ESRP2 | 1295 | LPIN2 | 1926 | SDC4 |
| 34 | ACY3 | 665 | ESYT2 | 1296 | LRCH2 | 1927 | SDHAP1 |
| 35 | ADAM15 | 666 | ETFB | 1297 | LRIG3 | 1928 | SDHAP3 |
| 36 | ADAM1A | 667 | ETFBKMT | 1298 | LRP1 | 1929 | SDK1 |
| 37 | ADAM9 | 668 | ETV1 | 1299 | LRP10 | 1930 | SDK2 |
| 38 | ADAMTS6 | 669 | EVA1A | 1300 | LRP11 | 1931 | SDR16C5 |
| 39 | ADAT1 | 670 | EVADR | 1301 | LRP3 | 1932 | SDR42E1 |
| 40 | ADCY1 | 671 | EXOC3 | 1302 | LRP4 | 1933 | SEC14L1P1 |
| 41 | ADCY9 | 672 | EXOC3L1 | 1303 | LRRC19 | 1934 | SEC16A |
| 42 | ADD1 | 673 | EXTL3 | 1304 | LRRC29 | 1935 | SEC24A |
| 43 | ADGRA3 | 674 | F11R | 1305 | LRRC61 | 1936 | SEC24D |
| 44 | ADGRF1 | 675 | F2RL1 | 1306 | LRRC75A-AS1 | 1937 | SEC31A |
| 45 | ADGRF4 | 676 | F3 | 1307 | LRRC8A | 1938 | SEC61G |
| 46 | ADGRF5 | 677 | FA2H | 1308 | LRRTM2 | 1939 | SEL1L3 |
| 47 | ADGRG1 | 678 | FAAH | 1309 | LRSAM1 | 1940 | SEMA3A |
| 48 | ADGRG2 | 679 | FAAH2 | 1310 | LTBP2 | 1941 | SEMA3C |
| 49 | ADGRG6 | 680 | FABP6 | 1311 | LTF | 1942 | SEMA3E |
| 50 | ADGRL1 | 681 | FAF2 | 1312 | LUC7L | 1943 | SEMA4B |
| 51 | ADGRL2 | 682 | FAHD1 | 1313 | LUCAT1 | 1944 | SEMA4F |
| 52 | ADGRV1 | 683 | FAM110C | 1314 | LURAP1L | 1945 | SENP8 |
| 53 | ADH1C | 684 | FAM114A1 | 1315 | LY6D | 1946 | SEPHS2 |
| 54 | ADHFE1 | 685 | FAM114A2 | 1316 | LY6E | 1947 | SEPN1 |
| 55 | ADM | 686 | FAM120C | 1317 | LY75 | 1948 | SEPT7P2 |
| 56 | AFAP1 | 687 | FAM127A | 1318 | LYNX1 | 1949 | SEPT8 |
| 57 | AFAP1-AS1 | 688 | FAM127B | 1319 | LYPD1 | 1950 | SERINC2 |
| 58 | AFDN | 689 | FAM127C | 1320 | LYPD3 | 1951 | SERINC3 |
| 59 | AFF4 | 690 | FAM135A | 1321 | MAATS1 | 1952 | SERINC5 |
| 60 | AGA | 691 | FAM13A | 1322 | MAEA | 1953 | SERPINA1 |
| 61 | AGAP5 | 692 | FAM13A-AS1 | 1323 | MAFTRR | 1954 | SERPINA3 |
| 62 | AGAP6 | 693 | FAM153C | 1324 | MAGED1 | 1955 | SERPINA5 |
| 63 | AGFG2 | 694 | FAM160A1 | 1325 | MAGED2 | 1956 | SERPINB3 |
| 64 | AGGF1 | 695 | FAM160B1 | 1326 | MAGEH1 | 1957 | SERPINE1 |
| 65 | AGR2 | 696 | FAM169A | 1327 | MAGT1 | 1958 | SERPINE2 |
| 66 | AGT | 697 | FAM174A | 1328 | MALSU1 | 1959 | SERPINH1 |
| 67 | AGTRAP | 698 | FAM174B | 1329 | MAN2A1 | 1960 | SEZ6L2 |
| 68 | AHCY | 699 | FAM177B | 1330 | MANSC1 | 1961 | SF3B3 |
| 69 | AHI1 | 700 | FAM193B | 1331 | MAOA | 1962 | SFRP1 |
| 70 | AHNAK2 | 701 | FAM198B | 1332 | MAP1B | 1963 | SFTA3 |
| 71 | AIDA | 702 | FAM200A | 1333 | MAP3K1 | 1964 | SFTPB |
| 72 | AIG1 | 703 | FAM20A | 1334 | MAP3K6 | 1965 | SGCE |
| 73 | AK3 | 704 | FAM21A | 1335 | MAP7D2 | 1966 | SGF29 |
| 74 | AK4 | 705 | FAM21EP | 1336 | MAP9 | 1967 | SGK223 |
| 75 | AK9 | 706 | FAM234A | 1337 | MAPK8IP1 | 1968 | SGPP2 |
| 76 | AKAP1 | 707 | FAM234B | 1338 | MAPRE2 | 1969 | SH2D4A |
| 77 | AKAP6 | 708 | FAM3C | 1339 | 6-Mar | 1970 | SH3BP2 |
| 78 | AKR1B1 | 709 | FAM47E | 1340 | 9-Mar | 1971 | SH3GL1 |
| 79 | AKR1B10 | 710 | FAM53A | 1341 | MARK1 | 1972 | SH3PXD2B |
| 80 | AKR1C1 | 711 | FAM60A | 1342 | MARK4 | 1973 | SHB |
| 81 | AKR1C2 | 712 | FAM69B | 1343 | MARS | 1974 | SHISA2 |
| 82 | AKR1C3 | 713 | FAM76A | 1344 | MARVELD1 | 1975 | SHISA9 |
| 83 | ALDH18A1 | 714 | FAM81B | 1345 | MARVELD3 | 1976 | SHMT2 |
| 84 | ALDH1A3 | 715 | FAM83A | 1346 | MAVS | 1977 | SHPK |
| 85 | ALDH7A1 | 716 | FAM86C2P | 1347 | MB | 1978 | SHROOM1 |
| 86 | ALDOA | 717 | FAM86DP | 1348 | MBD6 | 1979 | SIDT1 |
| 87 | ALG3 | 718 | FAM86EP | 1349 | MBNL2 | 1980 | SIL1 |
| 88 | ALG9 | 719 | FAM89A | 1350 | MBOAT1 | 1981 | SIPA1 |
| 89 | ALOX15B | 720 | FAM92A1 | 1351 | MBOAT2 | 1982 | SIRT3 |
| 90 | AMFR | 721 | FAM95C | 1352 | MBOAT7 | 1983 | SIX5 |
| 91 | AMIGO2 | 722 | FAP | 1353 | MBTD1 | 1984 | SKP1 |
| 92 | AMPH | 723 | FARP1 | 1354 | MBTPS1 | 1985 | SLC10A3 |
| 93 | AMT | 724 | FASN | 1355 | MCFD2 | 1986 | SLC12A2 |
| 94 | AMY2B | 725 | FASTK | 1356 | MCM9 | 1987 | SLC12A4 |
| 95 | AMZ2 | 726 | FAT1 | 1357 | MCOLN3 | 1988 | SLC12A7 |
| 96 | ANG | 727 | FAXDC2 | 1358 | MCRIP1 | 1989 | SLC13A3 |
| 97 | ANGPTL4 | 728 | FBLN2 | 1359 | MCRIP2 | 1990 | SLC16A12 |
| 98 | ANKAR | 729 | FBXL2 | 1360 | MCTS1 | 1991 | SLC16A14 |
| 99 | ANKDD1B | 730 | FBXL5 | 1361 | MDK | 1992 | SLC16A2 |
| 100 | ANKIB1 | 731 | FBXO31 | 1362 | MDM1 | 1993 | SLC16A7 |
| 101 | ANKMY2 | 732 | FBXO32 | 1363 | MDM2 | 1994 | SLC16A8 |
| 102 | ANKRA2 | 733 | FDPSP2 | 1364 | MED14 | 1995 | SLC16A9 |
| 103 | ANKRD18A | 734 | FER | 1365 | MED7 | 1996 | SLC22A17 |
| 104 | ANKRD19P | 735 | FER1L4 | 1366 | MED9 | 1997 | SLC22A18 |
| 105 | ANKRD20A12P | 736 | FEZF1 | 1367 | MEDAG | 1998 | SLC22A4 |
| 106 | ANKRD36B | 737 | FGA | 1368 | MEGF10 | 1999 | SLC25A18 |
| 107 | ANKRD36BP1 | 738 | FGB | 1369 | MEGF8 | 2000 | SLC25A21 |
| 108 | ANO3 | 739 | FGD1 | 1370 | MEIS3P1 | 2001 | SLC25A37 |
| 109 | ANO5 | 740 | FGD6 | 1371 | MELTF | 2002 | SLC25A4 |
| 110 | ANO6 | 741 | FGF13 | 1372 | MEPE | 2003 | SLC25A43 |
| 111 | ANOS1 | 742 | FGFBP1 | 1373 | MET | 2004 | SLC25A46 |
| 112 | ANTXR2 | 743 | FGG | 1374 | METTL1 | 2005 | SLC26A2 |
| 113 | ANXA2 | 744 | FGL1 | 1375 | METTL21B | 2006 | SLC2A1 |
| 114 | ANXA3 | 745 | FHL2 | 1376 | METTL26 | 2007 | SLC2A12 |
| 115 | ANXA5 | 746 | FHOD1 | 1377 | MFAP3 | 2008 | SLC2A4RG |
| 116 | AP1G1 | 747 | FHOD3 | 1378 | MFGE8 | 2009 | SLC30A5 |
| 117 | AP4S1 | 748 | FKBP11 | 1379 | MFSD2A | 2010 | SLC34A2 |
| 118 | AP5S1 | 749 | FKBP14 | 1380 | MFSD3 | 2011 | SLC35A4 |
| 119 | APBA2 | 750 | FKBP5 | 1381 | MFSD4A | 2012 | SLC35B4 |
| 120 | APBB3 | 751 | FKBP7 | 1382 | MFSD8 | 2013 | SLC35C1 |
| 121 | APC | 752 | FKBP9 | 1383 | MGAT4B | 2014 | SLC35C2 |
| 122 | APLP2 | 753 | FLJ10038 | 1384 | MGC32805 | 2015 | SLC35E2 |
| 123 | APMAP | 754 | FLJ20021 | 1385 | MGST1 | 2016 | SLC35E3 |
| 124 | APOBEC2 | 755 | FLJ22447 | 1386 | MGST2 | 2017 | SLC37A3 |
| 125 | APOD | 756 | FLJ23867 | 1387 | MICAL2 | 2018 | SLC37A4 |
| 126 | APOOL | 757 | FLJ32255 | 1388 | MICAL3 | 2019 | SLC38A10 |
| 127 | APTR | 758 | FLJ37453 | 1389 | MID1 | 2020 | SLC38A11 |
| 128 | AQP1 | 759 | FLNA | 1390 | MID2 | 2021 | SLC38A7 |
| 129 | ARAP3 | 760 | FLNB | 1391 | MIGA1 | 2022 | SLC39A11 |
| 130 | ARF5 | 761 | FLRT2 | 1392 | MIGA2 | 2023 | SLC39A13 |
| 131 | ARFGAP1 | 762 | FLRT3 | 1393 | MINPP1 | 2024 | SLC43A3 |
| 132 | ARFGEF2 | 763 | FLYWCH2 | 1394 | MKL2 | 2025 | SLC44A4 |
| 133 | ARFRP1 | 764 | FMC1 | 1395 | MKRN2OS | 2026 | SLC48A1 |
| 134 | ARHGAP10 | 765 | FN1 | 1396 | MLLT1 | 2027 | SLC6A11 |
| 135 | ARHGAP17 | 766 | FNDC10 | 1397 | MLXIP | 2028 | SLC6A3 |
| 136 | ARHGAP23 | 767 | FNDC3A | 1398 | MMD | 2029 | SLC7A2 |
| 137 | ARHGAP24 | 768 | FNDC4 | 1399 | MMP1 | 2030 | SLC9A3R2 |
| 138 | ARHGAP29 | 769 | FOXF2 | 1400 | MMP13 | 2031 | SLC9A5 |
| 139 | ARHGAP40 | 770 | FOXK1 | 1401 | MMP15 | 2032 | SLC9A7 |
| 140 | ARHGAP5 | 771 | FOXP2 | 1402 | MMP28 | 2033 | SLCO2A1 |
| 141 | ARHGAP5-AS1 | 772 | FOXQ1 | 1403 | MMP7 | 2034 | SLCO3A1 |
| 142 | ARHGEF12 | 773 | FRG1HP | 1404 | MOCS1 | 2035 | SLCO4C1 |
| 143 | ARHGEF26-AS1 | 774 | FRMD5 | 1405 | MOCS2 | 2036 | SLED1 |
| 144 | ARHGEF33 | 775 | FRMD6 | 1406 | MON1A | 2037 | SLF1 |
| 145 | ARHGEF35 | 776 | FRS2 | 1407 | MON2 | 2038 | SLIT2 |
| 146 | ARHGEF38 | 777 | FSIP2 | 1408 | MPG | 2039 | SLITRK6 |
| 147 | ARHGEF38-IT1 | 778 | FSTL3 | 1409 | MPRIP | 2040 | SLPI |
| 148 | ARL1 | 779 | FSTL4 | 1410 | MPZL2 | 2041 | SLX4IP |
| 149 | ARL14 | 780 | FUCA1 | 1411 | MR1 | 2042 | SMA5 |
| 150 | ARL16 | 781 | FURIN | 1412 | MRM2 | 2043 | SMAD1 |
| 151 | ARL2BP | 782 | FUT2 | 1413 | MRM3 | 2044 | SMAD5 |
| 152 | ARL4A | 783 | FUT3 | 1414 | MROH1 | 2045 | SMARCA1 |
| 153 | ARL4D | 784 | FXYD3 | 1415 | MRPL22 | 2046 | SMC1B |
| 154 | ARMCX4 | 785 | FYCO1 | 1416 | MRPL3 | 2047 | SMIM1 |
| 155 | ARPC1A | 786 | FZD1 | 1417 | MRPL32 | 2048 | SMIM10L2A |
| 156 | ARRDC2 | 787 | FZD2 | 1418 | MRPL38 | 2049 | SMIM14 |
| 157 | ARRDC3 | 788 | FZD6 | 1419 | MRPL53 | 2050 | SMIM22 |
| 158 | ARSD | 789 | G3BP1 | 1420 | MRPL57 | 2051 | SMIM24 |
| 159 | ARSE | 790 | GABARAP | 1421 | MRPL58 | 2052 | SMIM3 |
| 160 | ARSJ | 791 | GABARAPL2 | 1422 | MRPS17 | 2053 | SMPD1 |
| 161 | ARX | 792 | GABRE | 1423 | MRPS34 | 2054 | SMPD2 |
| 162 | ASAP3 | 793 | GADD45B | 1424 | MRPS36 | 2055 | SNAI2 |
| 163 | ASB16-AS1 | 794 | GADD45G | 1425 | MRS2P2 | 2056 | SNCAIP |
| 164 | ASCC3 | 795 | GALNT10 | 1426 | MSH3 | 2057 | SNCG |
| 165 | ASPH | 796 | GALNT12 | 1427 | MSRB1 | 2058 | SNHG11 |
| 166 | ATAD1 | 797 | GALNT14 | 1428 | MST1P2 | 2059 | SNHG17 |
| 167 | ATAD3C | 798 | GALT | 1429 | MST1R | 2060 | SNHG18 |
| 168 | ATG101 | 799 | GAREM2 | 1430 | MT1DP | 2061 | SNHG20 |
| 169 | ATG9B | 800 | GATAD1 | 1431 | MT1E | 2062 | SNHG3 |
| 170 | ATMIN | 801 | GATS | 1432 | MT1F | 2063 | SNORA58 |
| 171 | ATOH8 | 802 | GATSL2 | 1433 | MT1L | 2064 | SNRNP200 |
| 172 | ATP13A2 | 803 | GBP3 | 1434 | MT1M | 2065 | SNRNP48 |
| 173 | ATP13A4 | 804 | GCN1 | 1435 | MT1X | 2066 | SNTA1 |
| 174 | ATP13A4-AS1 | 805 | GCNT1 | 1436 | MT2A | 2067 | SNTB2 |
| 175 | ATP1A1-AS1 | 806 | GCSH | 1437 | MTFP1 | 2068 | SNX12 |
| 176 | ATP23 | 807 | GEMIN5 | 1438 | MTHFSD | 2069 | SNX13 |
| 177 | ATP2B4 | 808 | GEMIN8 | 1439 | MTMR9LP | 2070 | SNX18 |
| 178 | ATP2C2 | 809 | GET4 | 1440 | MTUS1 | 2071 | SNX19 |
| 179 | ATP5SL | 810 | GFAP | 1441 | MTX3 | 2072 | SNX21 |
| 180 | ATP6V0C | 811 | GFPT2 | 1442 | MUC13 | 2073 | SOGA3 |
| 181 | ATP6V0E1 | 812 | GGT3P | 1443 | MUC15 | 2074 | SORD |
| 182 | ATP6V0E2 | 813 | GGT5 | 1444 | MUC16 | 2075 | SORT1 |
| 183 | ATP9A | 814 | GHDC | 1445 | MUC20 | 2076 | SP2-AS1 |
| 184 | ATXN2L | 815 | GJB2 | 1446 | MUC4 | 2077 | SPACA9 |
| 185 | AVIL | 816 | GJB3 | 1447 | MUC5B | 2078 | SPAG1 |
| 186 | AVL9 | 817 | GJC3 | 1448 | MUL1 | 2079 | SPAG4 |
| 187 | AZGP1 | 818 | GLB1 | 1449 | MUM1L1 | 2080 | SPARC |
| 188 | B3GALNT1 | 819 | GLB1L3 | 1450 | MUT | 2081 | SPATA6 |
| 189 | B3GALT6 | 820 | GLDN | 1451 | MVP | 2082 | SPDEF |
| 190 | B4GALNT1 | 821 | GLG1 | 1452 | MXRA7 | 2083 | SPEF2 |
| 191 | B4GALT1 | 822 | GLIDR | 1453 | MYDGF | 2084 | SPESP1 |
| 192 | B4GALT7 | 823 | GLIS2 | 1454 | MYEOV | 2085 | SPIDR |
| 193 | B9D1 | 824 | GLRB | 1455 | MYH14 | 2086 | SPIN4 |
| 194 | BACE1 | 825 | GLTP | 1456 | MYO10 | 2087 | SPINK1 |
| 195 | BACE2 | 826 | GNAI1 | 1457 | MYO1D | 2088 | SPINK13 |
| 196 | BAIAP2L1 | 827 | GNAL | 1458 | MYO1E | 2089 | SPINK2 |
| 197 | BBOF1 | 828 | GNAS | 1459 | MYO5C | 2090 | SPINK5 |
| 198 | BBOX1 | 829 | GNG12 | 1460 | MYOF | 2091 | SPINT1 |
| 199 | BBS1 | 830 | GNPTG | 1461 | NAA20 | 2092 | SPINT2 |
| 200 | BBS12 | 831 | GNS | 1462 | NAALADL2 | 2093 | SPOCK1 |
| 201 | BBS2 | 832 | GOLGA1 | 1463 | NAB2 | 2094 | SPOCK3 |
| 202 | BBX | 833 | GOLGA2P7 | 1464 | NAMPT | 2095 | SPRED1 |
| 203 | BCAP31 | 834 | GOLGA6L5P | 1465 | NANP | 2096 | SPRED2 |
| 204 | BCAR1 | 835 | GOLGA8A | 1466 | NAPSA | 2097 | SPRR1B |
| 205 | BCAR3 | 836 | GOLM1 | 1467 | NAV1 | 2098 | SPRY1 |
| 206 | BCDIN3D | 837 | GON7 | 1468 | NAXD | 2099 | SPRY4 |
| 207 | BCKDHA | 838 | GOPC | 1469 | NAXE | 2100 | SPRY4-IT1 |
| 208 | BCKDK | 839 | GOT2 | 1470 | NBPF15 | 2101 | SPTB |
| 209 | BCL2L1 | 840 | GPAT2 | 1471 | NCKAP1 | 2102 | SPTLC1 |
| 210 | BCL2L15 | 841 | GPAT3 | 1472 | NCOA5 | 2103 | SQSTM1 |
| 211 | BCS1L | 842 | GPC3 | 1473 | NCOA6 | 2104 | SRA1 |
| 212 | BDH2 | 843 | GPC6 | 1474 | NDFIP1 | 2105 | SRD5A1 |
| 213 | BEAN1 | 844 | GPCPD1 | 1475 | NDOR1 | 2106 | SRD5A2 |
| 214 | BEND5 | 845 | GPM6B | 1476 | NDP | 2107 | SRD5A3 |
| 215 | BEX2 | 846 | GPR12 | 1477 | NDRG4 | 2108 | SREBF1 |
| 216 | BEX3 | 847 | GPR161 | 1478 | NDUFA2 | 2109 | SRFBP1 |
| 217 | BFSP1 | 848 | GPR22 | 1479 | NDUFA7 | 2110 | SRGAP1 |
| 218 | BICC1 | 849 | GPR37 | 1480 | NDUFAB1 | 2111 | SRPRA |
| 219 | BICDL2 | 850 | GPR39 | 1481 | NDUFAF2 | 2112 | SRPX2 |
| 220 | BIVM | 851 | GPR87 | 1482 | NDUFB10 | 2113 | SRSF6 |
| 221 | BLCAP | 852 | GPRC5C | 1483 | NDUFC1 | 2114 | SRSF8 |
| 222 | BLID | 853 | GPX3 | 1484 | NDUFS4 | 2115 | SRXN1 |
| 223 | BMF | 854 | GPX7 | 1485 | NECTIN1 | 2116 | SSBP2 |
| 224 | BMP1 | 855 | GPX8 | 1486 | NECTIN2 | 2117 | SSH3 |
| 225 | BMP3 | 856 | GRAMD1C | 1487 | NECTIN3 | 2118 | SSR4 |
| 226 | BMP8A | 857 | GRAMD3 | 1488 | NECTIN4 | 2119 | ST14 |
| 227 | BMS1P21 | 858 | GRB10 | 1489 | NEMP1 | 2120 | ST3GAL5 |
| 228 | BMS1P5 | 859 | GREB1 | 1490 | NET1 | 2121 | ST8SIA4 |
| 229 | BMT2 | 860 | GRHL1 | 1491 | NETO2 | 2122 | STAC |
| 230 | BOK | 861 | GRIK2 | 1492 | NEURL1B | 2123 | STAG3 |
| 231 | BOLA1 | 862 | GRIN2C | 1493 | NFAT5 | 2124 | STAG3L2 |
| 232 | BPIFA1 | 863 | GRIP1 | 1494 | NFATC3 | 2125 | STARD10 |
| 233 | BPIFA2 | 864 | GRM4 | 1495 | NFIB | 2126 | STARD4-AS1 |
| 234 | BRAT1 | 865 | GSE1 | 1496 | NFXL1 | 2127 | STAT6 |
| 235 | BRCC3 | 866 | GSS | 1497 | NFYC-AS1 | 2128 | STAU1 |
| 236 | BRPF3 | 867 | GSTK1 | 1498 | NGEF | 2129 | STBD1 |
| 237 | BSG | 868 | GSTT1 | 1499 | NGRN | 2130 | STC1 |
| 238 | BTBD11 | 869 | GTF2H2B | 1500 | NHEJ1 | 2131 | STEAP1 |
| 239 | BTC | 870 | GTF2IRD1 | 1501 | NHP2 | 2132 | STEAP2 |
| 240 | BVES | 871 | GTF3C1 | 1502 | NHS | 2133 | STEAP3 |
| 241 | C10orf25 | 872 | GTPBP10 | 1503 | NIM1K | 2134 | STK32A |
| 242 | C11orf57 | 873 | GUCY1A3 | 1504 | NINL | 2135 | STMN3 |
| 243 | C11orf70 | 874 | GUCY2D | 1505 | NIPAL1 | 2136 | STON2 |
| 244 | C11orf71 | 875 | GUSBP4 | 1506 | NIPAL2 | 2137 | STOX1 |
| 245 | C11orf80 | 876 | GYG2 | 1507 | NIPBL-AS1 | 2138 | STRA6 |
| 246 | C11orf91 | 877 | H2AFJ | 1508 | NIPSNAP1 | 2139 | STRADA |
| 247 | C12orf66 | 878 | HABP2 | 1509 | NKAPP1 | 2140 | STRN4 |
| 248 | C14orf119 | 879 | HACD3 | 1510 | NKIRAS2 | 2141 | STT3A |
| 249 | C15orf62 | 880 | HAR1B | 1511 | NME5 | 2142 | STX16 |
| 250 | C16orf45 | 881 | HARS | 1512 | NME6 | 2143 | STX1A |
| 251 | C16orf46 | 882 | HAS2 | 1513 | NMNAT2 | 2144 | STYXL1 |
| 252 | C16orf58 | 883 | HBQ1 | 1514 | NMRAL1 | 2145 | SUFU |
| 253 | C16orf62 | 884 | HCG11 | 1515 | NNMT | 2146 | SUGCT |
| 254 | C16orf91 | 885 | HCG26 | 1516 | NNT | 2147 | SULF1 |
| 255 | C16orf95 | 886 | HCG4 | 1517 | NOB1 | 2148 | SULF2 |
| 256 | C17orf51 | 887 | HCP5 | 1518 | NOL3 | 2149 | SULT2B1 |
| 257 | C19orf70 | 888 | HDAC8 | 1519 | NOL4L | 2150 | SULT4A1 |
| 258 | C1GALT1C1 | 889 | HDX | 1520 | NOMO1 | 2151 | SUMF1 |
| 259 | C1orf106 | 890 | HEATR5A | 1521 | NOP14-AS1 | 2152 | SUN1 |
| 260 | C1orf115 | 891 | HELQ | 1522 | NOP16 | 2153 | SV2A |
| 261 | C1QTNF1 | 892 | HELZ2 | 1523 | NORAD | 2154 | SYBU |
| 262 | C1QTNF3 | 893 | HEMK1 | 1524 | NOTCH2NL | 2155 | SYCE1L |
| 263 | C1R | 894 | HENMT1 | 1525 | NOTCH3 | 2156 | SYNDIG1L |
| 264 | C1S | 895 | HERC2 | 1526 | NOV | 2157 | SYNGR3 |
| 265 | C20orf197 | 896 | HERC2P7 | 1527 | NOX1 | 2158 | SYNPO |
| 266 | C21orf33 | 897 | HES2 | 1528 | NPAS3 | 2159 | SYNPO2 |
| 267 | C2orf74 | 898 | HEXB | 1529 | NPDC1 | 2160 | SYPL1 |
| 268 | C3 | 899 | HEXDC | 1530 | NPEPPS | 2161 | SYT12 |
| 269 | C3orf36 | 900 | HHATL | 1531 | NPHP1 | 2162 | SYTL2 |
| 270 | C4orf48 | 901 | HHIPL2 | 1532 | NPIPB11 | 2163 | SYTL4 |
| 271 | C5orf15 | 902 | HHLA2 | 1533 | NPIPB15 | 2164 | SYTL5 |
| 272 | C5orf24 | 903 | HIBADH | 1534 | NPNT | 2165 | T |
| 273 | C5orf66 | 904 | HID1 | 1535 | NPR3 | 2166 | TACSTD2 |
| 274 | C6orf106 | 905 | HIKESHI | 1536 | NPTN | 2167 | TAF7 |
| 275 | C6orf89 | 906 | HILPDA | 1537 | NPTX2 | 2168 | TAF9B |
| 276 | C7orf13 | 907 | HIP1 | 1538 | NQO1 | 2169 | TAGLN2 |
| 277 | C8B | 908 | HIP1R | 1539 | NR2F1 | 2170 | TANGO6 |
| 278 | C8orf4 | 909 | HIPK2 | 1540 | NR2F1-AS1 | 2171 | TAPBP |
| 279 | C9orf116 | 910 | HIST1H2AE | 1541 | NR2F6 | 2172 | TAPBPL |
| 280 | CADPS | 911 | HIST1H2BK | 1542 | NREP | 2173 | TBC1D10A |
| 281 | CALD1 | 912 | HIST1H4J | 1543 | NRK | 2174 | TBC1D10B |
| 282 | CALU | 913 | HKR1 | 1544 | NRP1 | 2175 | TBC1D20 |
| 283 | CAMK2D | 914 | HLA-DOA | 1545 | NRP2 | 2176 | TBC1D2B |
| 284 | CAMK2G | 915 | HLA-DRB5 | 1546 | NRSN2 | 2177 | TBC1D30 |
| 285 | CAMK2N1 | 916 | HLA-G | 1547 | NSF | 2178 | TBC1D32 |
| 286 | CAND1 | 917 | HLA-H | 1548 | NSMCE1 | 2179 | TBC1D8 |
| 287 | CANT1 | 918 | HLA-J | 1549 | NSUN5P1 | 2180 | TBC1D9 |
| 288 | CANX | 919 | HLA-L | 1550 | NT5DC3 | 2181 | TBC1D9B |
| 289 | CAPN13 | 920 | HLTF | 1551 | NT5E | 2182 | TBK1 |
| 290 | CAPN2 | 921 | HM13 | 1552 | NTHL1 | 2183 | TBL2 |
| 291 | CAPN6 | 922 | HMGB3 | 1553 | NTN4 | 2184 | TBL3 |
| 292 | CAPN8 | 923 | HMGN5 | 1554 | NUCB2 | 2185 | TBP |
| 293 | CARD10 | 924 | HMOX2 | 1555 | NUDCD2 | 2186 | TBRG4 |
| 294 | CARD14 | 925 | HN1L | 1556 | NUDCD3 | 2187 | TCAF1 |
| 295 | CARD19 | 926 | HOMER3 | 1557 | NUDT13 | 2188 | TCAM1P |
| 296 | CARD6 | 927 | HOOK1 | 1558 | NUDT16L1 | 2189 | TCEAL4 |
| 297 | CASC4 | 928 | HOPX | 1559 | NUDT16P1 | 2190 | TCEAL9 |
| 298 | CASC9 | 929 | HOXB7 | 1560 | NUDT9 | 2191 | TCERG1 |
| 299 | CASK | 930 | HP | 1561 | NUP107 | 2192 | TCN1 |
| 300 | CASP10 | 931 | HPF1 | 1562 | NUP62CL | 2193 | TCTA |
| 301 | CASP6 | 932 | HPGD | 1563 | OAS1 | 2194 | TCTN3 |
| 302 | CASP7 | 933 | HS2ST1 | 1564 | OAS3 | 2195 | TDRD10 |
| 303 | CAST | 934 | HSBP1 | 1565 | OAT | 2196 | TDRD7 |
| 304 | CAV2 | 935 | HSD17B10 | 1566 | OBSL1 | 2197 | TECR |
| 305 | CBFA2T2 | 936 | HSD17B4 | 1567 | OCIAD2 | 2198 | TEFM |
| 306 | CBY3 | 937 | HSD3B7 | 1568 | OCLN | 2199 | TEKT4P2 |
| 307 | CCBE1 | 938 | HSP90AB4P | 1569 | OCRL | 2200 | TENM4 |
| 308 | CCDC113 | 939 | HSPA4L | 1570 | OFD1 | 2201 | TET1 |
| 309 | CCDC126 | 940 | HSPA9 | 1571 | OLMALINC | 2202 | TEX264 |
| 310 | CCDC127 | 941 | HSPB1 | 1572 | OPHN1 | 2203 | TEX9 |
| 311 | CCDC142 | 942 | HTR3A | 1573 | OS9 | 2204 | TFAP2A |
| 312 | CCDC144A | 943 | HTRA1 | 1574 | OSBP | 2205 | TFAP2D |
| 313 | CCDC144B | 944 | HTT | 1575 | OSBPL6 | 2206 | TFPI |
| 314 | CCDC148 | 945 | HUWE1 | 1576 | OSMR | 2207 | TGFA |
| 315 | CCDC160 | 946 | HYAL2 | 1577 | OSTM1 | 2208 | TGFBI |
| 316 | CCDC171 | 947 | HYDIN | 1578 | OXT | 2209 | TGFBRAP1 |
| 317 | CCDC186 | 948 | ID1 | 1579 | P2RY6 | 2210 | TGM2 |
| 318 | CCDC18-AS1 | 949 | IDO1 | 1580 | P3H4 | 2211 | TGOLN2 |
| 319 | CCDC192 | 950 | IDS | 1581 | P4HA2 | 2212 | THAP12 |
| 320 | CCDC43 | 951 | IFI27 | 1582 | P4HA2-AS1 | 2213 | THAP6 |
| 321 | CCDC7 | 952 | IFITM1 | 1583 | P4HA3 | 2214 | THBS1 |
| 322 | CCDC71L | 953 | IFITM10 | 1584 | P4HB | 2215 | THBS3 |
| 323 | CCK | 954 | IFITM3 | 1585 | PA2G4P4 | 2216 | THTPA |
| 324 | CCL28 | 955 | IFNGR1 | 1586 | PAEP | 2217 | TIGD6 |
| 325 | CCND1 | 956 | IFT122 | 1587 | PAFAH2 | 2218 | TIMM17B |
| 326 | CCNG2 | 957 | IFT140 | 1588 | PAGR1 | 2219 | TIMP1 |
| 327 | CCNJL | 958 | IFT43 | 1589 | PAIP1 | 2220 | TIMP2 |
| 328 | CCNO | 959 | IFT81 | 1590 | PAK3 | 2221 | TIMP3 |
| 329 | CCS | 960 | IGF1R | 1591 | PALB2 | 2222 | TK2 |
| 330 | CCSER1 | 961 | IGF2BP2 | 1592 | PAM | 2223 | TKFC |
| 331 | CCT2 | 962 | IGF2R | 1593 | PANX2 | 2224 | TKT |
| 332 | CCT6P1 | 963 | IGFBP4 | 1594 | PAPLN | 2225 | TLE1 |
| 333 | CCT6P3 | 964 | IGFBP5 | 1595 | PAPSS2 | 2226 | TLE2 |
| 334 | CD109 | 965 | IGFBP6 | 1596 | PARD6B | 2227 | TLR5 |
| 335 | CD164L2 | 966 | IGFBPL1 | 1597 | PARM1 | 2228 | TM4SF18 |
| 336 | CD276 | 967 | IGSF3 | 1598 | PARP3 | 2229 | TM4SF1-AS1 |
| 337 | CD38 | 968 | IK | 1599 | PARTICL | 2230 | TM9SF1 |
| 338 | CD40 | 969 | IL13RA1 | 1600 | PARVA | 2231 | TM9SF2 |
| 339 | CD46 | 970 | IL15 | 1601 | PATJ | 2232 | TM9SF4 |
| 340 | CD59 | 971 | IL1R1 | 1602 | PAX8 | 2233 | TMBIM1 |
| 341 | CD74 | 972 | IL20RB | 1603 | PAX8-AS1 | 2234 | TMBIM4 |
| 342 | CD99L2 | 973 | IL36RN | 1604 | PAXIP1 | 2235 | TMC4 |
| 343 | CDA | 974 | IL4R | 1605 | PAXIP1-AS1 | 2236 | TMC5 |
| 344 | CDC20B | 975 | IL6ST | 1606 | PC | 2237 | TMCO3 |
| 345 | CDC25B | 976 | ILDR1 | 1607 | PCAT18 | 2238 | TMED1 |
| 346 | CDC37L1-AS1 | 977 | IMMP2L | 1608 | PCBD1 | 2239 | TMED3 |
| 347 | CDC42BPA | 978 | INA | 1609 | PCDH1 | 2240 | TMED7 |
| 348 | CDC42EP1 | 979 | INPP4B | 1610 | PCDH7 | 2241 | TMEM104 |
| 349 | CDC42EP5 | 980 | INTS1 | 1611 | PCDHB10 | 2242 | TMEM106B |
| 350 | CDCP1 | 981 | INTS5 | 1612 | PCDHB12 | 2243 | TMEM129 |
| 351 | CDH1 | 982 | INTU | 1613 | PCDHB14 | 2244 | TMEM132A |
| 352 | CDH11 | 983 | IP6K3 | 1614 | PCDHB19P | 2245 | TMEM133 |
| 353 | CDH15 | 984 | IPP | 1615 | PCDHB4 | 2246 | TMEM14A |
| 354 | CDH3 | 985 | IPW | 1616 | PCDHGA10 | 2247 | TMEM161B |
| 355 | CDIPT | 986 | IQCA1 | 1617 | PCGF3 | 2248 | TMEM161B-AS1 |
| 356 | CDK14 | 987 | IQCK | 1618 | PCLO | 2249 | TMEM165 |
| 357 | CDK2AP1 | 988 | IRAK3 | 1619 | PCMTD2 | 2250 | TMEM167A |
| 358 | CDK4 | 989 | IRX3 | 1620 | PCNP | 2251 | TMEM173 |
| 359 | CDK5RAP1 | 990 | ISCA2 | 1621 | PCNX3 | 2252 | TMEM175 |
| 360 | CDK7 | 991 | ISM1 | 1622 | PCOLCE2 | 2253 | TMEM185A |
| 361 | CDKL3 | 992 | ISOC1 | 1623 | PCP4L1 | 2254 | TMEM186 |
| 362 | CDKN2A | 993 | IST1 | 1624 | PCSK1N | 2255 | TMEM191A |
| 363 | CDKN2AIPNL | 994 | ITFG1 | 1625 | PDGFC | 2256 | TMEM199 |
| 364 | CDKN2B | 995 | ITGA1 | 1626 | PDGFRL | 2257 | TMEM205 |
| 365 | CDS1 | 996 | ITGA2 | 1627 | PDIA3P1 | 2258 | TMEM208 |
| 366 | CEACAM1 | 997 | ITGA3 | 1628 | PDIA4 | 2259 | TMEM222 |
| 367 | CEACAM5 | 998 | ITGAV | 1629 | PDK3 | 2260 | TMEM230 |
| 368 | CEACAM6 | 999 | ITGB3 | 1630 | PDLIM1 | 2261 | TMEM231 |
| 369 | CELF2-AS2 | 1000 | ITGB4 | 1631 | PDLIM4 | 2262 | TMEM245 |
| 370 | CEMIP | 1001 | ITGB5 | 1632 | PDLIM5 | 2263 | TMEM263 |
| 371 | CENPBD1 | 1002 | ITGB6 | 1633 | PDLIM7 | 2264 | TMEM265 |
| 372 | CEP112 | 1003 | ITGB8 | 1634 | PDPK1 | 2265 | TMEM267 |
| 373 | CEP126 | 1004 | ITM2B | 1635 | PDPR | 2266 | TMEM268 |
| 374 | CEP44 | 1005 | ITPKA | 1636 | PDXDC1 | 2267 | TMEM30B |
| 375 | CES1 | 1006 | ITPKC | 1637 | PDXP | 2268 | TMEM35B |
| 376 | CETN2 | 1007 | ITPRIPL2 | 1638 | PDZD11 | 2269 | TMEM43 |
| 377 | CETN3 | 1008 | IVL | 1639 | PDZK1IP1 | 2270 | TMEM45B |
| 378 | CFAP20 | 1009 | JADE2 | 1640 | PELI3 | 2271 | TMEM5 |
| 379 | CFAP221 | 1010 | JAG1 | 1641 | PELO | 2272 | TMEM59 |
| 380 | CFAP53 | 1011 | JMJD4 | 1642 | PENK | 2273 | TMEM63C |
| 381 | CFAP69 | 1012 | JMJD7 | 1643 | PERP | 2274 | TMEM74B |
| 382 | CFAP97 | 1013 | KANSL3 | 1644 | PEX11G | 2275 | TMEM8A |
| 383 | CFB | 1014 | KAT14 | 1645 | PEX12 | 2276 | TMEM9 |
| 384 | CFDP1 | 1015 | KAT8 | 1646 | PFDN1 | 2277 | TMEM92 |
| 385 | CFH | 1016 | KBTBD12 | 1647 | PFKP | 2278 | TMEM92-AS1 |
| 386 | CFI | 1017 | KCCAT211 | 1648 | PFN1P2 | 2279 | TMEM94 |
| 387 | CFTR | 1018 | KCCAT333 | 1649 | PFN2 | 2280 | TMEM98 |
| 388 | CGN | 1019 | KCNA3 | 1650 | PGAM2 | 2281 | TMEM9B-AS1 |
| 389 | CGREF1 | 1020 | KCNE2 | 1651 | PGK1 | 2282 | TMPRSS11E |
| 390 | CH25H | 1021 | KCNJ1 | 1652 | PGM1 | 2283 | TMPRSS13 |
| 391 | CHD6 | 1022 | KCNK12 | 1653 | PGM2L1 | 2284 | TMPRSS4 |
| 392 | CHD9 | 1023 | KCNMB4 | 1654 | PGRMC1 | 2285 | TMTC3 |
| 393 | CHGB | 1024 | KCNN4 | 1655 | PGS1 | 2286 | TMTC4 |
| 394 | CHI3L1 | 1025 | KCNS3 | 1656 | PHACTR3 | 2287 | TMX4 |
| 395 | CHIC1 | 1026 | KCTD1 | 1657 | PHB | 2288 | TNC |
| 396 | CHID1 | 1027 | KCTD21 | 1658 | PHC2 | 2289 | TNFRSF12A |
| 397 | CHMP1A | 1028 | KDELC2 | 1659 | PHF12 | 2290 | TNFRSF1A |
| 398 | CHMP4C | 1029 | KDM5D | 1660 | PHKB | 2291 | TNFSF10 |
| 399 | CHPF | 1030 | KDR | 1661 | PHLDA2 | 2292 | TNNC2 |
| 400 | CHPF2 | 1031 | KIAA0141 | 1662 | PHOSPHO2 | 2293 | TNNI1 |
| 401 | CHST1 | 1032 | KIAA0232 | 1663 | PHYKPL | 2294 | TNRC6A |
| 402 | CHST15 | 1033 | KIAA0391 | 1664 | PI4KAP2 | 2295 | TNS3 |
| 403 | CHST3 | 1034 | KIAA0430 | 1665 | PIGG | 2296 | TNS4 |
| 404 | CHSY3 | 1035 | KIAA1107 | 1666 | PIGQ | 2297 | TOM1 |
| 405 | CIB1 | 1036 | KIAA1191 | 1667 | PIGT | 2298 | TOMM6 |
| 406 | CIDEC | 1037 | KIAA1211L | 1668 | PIH1D2 | 2299 | TOMM70 |
| 407 | CIDECP | 1038 | KIAA1217 | 1669 | PIK3R2 | 2300 | TOP1P1 |
| 408 | CISH | 1039 | KIAA1549 | 1670 | PIP | 2301 | TOX4 |
| 409 | CKAP4 | 1040 | KIAA1644 | 1671 | PIP4K2C | 2302 | TP53TG1 |
| 410 | CLCN3 | 1041 | KIAA1671 | 1672 | PITX1 | 2303 | TPBG |
| 411 | CLDN10 | 1042 | KIF16B | 1673 | PJA1 | 2304 | TPCN1 |
| 412 | CLDN10-AS1 | 1043 | KIF3B | 1674 | PJA2 | 2305 | TPCN2 |
| 413 | CLDN12 | 1044 | KIT | 1675 | PKD2 | 2306 | TPD52L1 |
| 414 | CLDN2 | 1045 | KITLG | 1676 | PKIB | 2307 | TPD52L2 |
| 415 | CLDN3 | 1046 | KIZ | 1677 | PKM | 2308 | TPM3P9 |
| 416 | CLDN4 | 1047 | KLF8 | 1678 | PLA2G10 | 2309 | TPPP |
| 417 | CLINT1 | 1048 | KLHDC1 | 1679 | PLA2G12A | 2310 | TPRN |
| 418 | CLK4 | 1049 | KLHDC10 | 1680 | PLAT | 2311 | TRABD2B |
| 419 | CLN3 | 1050 | KLHDC7A | 1681 | PLAU | 2312 | TRADD |
| 420 | CLSTN1 | 1051 | KLHL2 | 1682 | PLCB1 | 2313 | TRAPPC11 |
| 421 | CLUAP1 | 1052 | KLHL35 | 1683 | PLCG1-AS1 | 2314 | TRAPPC2B |
| 422 | CLUHP3 | 1053 | KLHL5 | 1684 | PLEC | 2315 | TRAPPC5 |
| 423 | CMAHP | 1054 | KLK6 | 1685 | PLEKHA7 | 2316 | TRDMT1 |
| 424 | CMBL | 1055 | KMT5B | 1686 | PLEKHB1 | 2317 | TREM1 |
| 425 | CMC4 | 1056 | KNOP1 | 1687 | PLEKHG1 | 2318 | TRIL |
| 426 | CMTM4 | 1057 | KRBA2 | 1688 | PLEKHG3 | 2319 | TRIM16 |
| 427 | CMTR2 | 1058 | KRBOX1 | 1689 | PLEKHG4 | 2320 | TRIM2 |
| 428 | CNDP2 | 1059 | KRT15 | 1690 | PLEKHH2 | 2321 | TRIM23 |
| 429 | CNFN | 1060 | KRT16 | 1691 | PLEKHM1P1 | 2322 | TRIM31 |
| 430 | CNIH3 | 1061 | KRT16P1 | 1692 | PLLP | 2323 | TRIM47 |
| 431 | CNN2 | 1062 | KRT16P2 | 1693 | PLOD1 | 2324 | TRIM52-AS1 |
| 432 | CNNM1 | 1063 | KRT16P3 | 1694 | PLOD3 | 2325 | TRIM7 |
| 433 | CNNM4 | 1064 | KRT17 | 1695 | PLPP2 | 2326 | TRIM9 |
| 434 | CNOT2 | 1065 | KRT18 | 1696 | PLPP3 | 2327 | TRIP6 |
| 435 | CNOT8 | 1066 | KRT19 | 1697 | PLPP4 | 2328 | TRPC1 |
| 436 | CNPY2 | 1067 | KRT6B | 1698 | PLPP5 | 2329 | TRPV1 |
| 437 | CNTNAP2 | 1068 | KRT7 | 1699 | PLPPR2 | 2330 | TSC2 |
| 438 | COG4 | 1069 | KRT8 | 1700 | PLS3 | 2331 | TSC22D1 |
| 439 | COG7 | 1070 | KRT80 | 1701 | PLXNA3 | 2332 | TSC22D3 |
| 440 | COL13A1 | 1071 | KRTCAP2 | 1702 | PLXNB1 | 2333 | TSFM |
| 441 | COL17A1 | 1072 | KRTCAP3 | 1703 | PLXNB2 | 2334 | TSKU |
| 442 | COL1A1 | 1073 | KYAT1 | 1704 | PLXND1 | 2335 | TSPAN1 |
| 443 | COL1A2 | 1074 | KYAT3 | 1705 | PMEPA1 | 2336 | TSPAN11 |
| 444 | COL21A1 | 1075 | L2HGDH | 1706 | PML | 2337 | TSPAN12 |
| 445 | COL4A5 | 1076 | LACAT8 | 1707 | PMM2 | 2338 | TSPAN3 |
| 446 | COL6A1 | 1077 | LACTB2-AS1 | 1708 | PMS2P3 | 2339 | TSPAN31 |
| 447 | COL6A2 | 1078 | LAD1 | 1709 | PNMA3 | 2340 | TSPAN6 |
| 448 | COL7A1 | 1079 | LAMA4 | 1710 | PNN | 2341 | TSPAN8 |
| 449 | COMT | 1080 | LAMA5 | 1711 | PNPO | 2342 | TSPYL5 |
| 450 | COMTD1 | 1081 | LAMB1 | 1712 | POC1B | 2343 | TTC30B |
| 451 | COQ9 | 1082 | LAMB2 | 1713 | POC5 | 2344 | TTC37 |
| 452 | COX11 | 1083 | LAMB3 | 1714 | PODN | 2345 | TTC3P1 |
| 453 | CP | 1084 | LAMC2 | 1715 | POF1B | 2346 | TTC9 |
| 454 | CPD | 1085 | LAMP2 | 1716 | POLD4 | 2347 | TTI1 |
| 455 | CPE | 1086 | LAPTM4B | 1717 | POMC | 2348 | TTLL7 |
| 456 | CPM | 1087 | LARGE1 | 1718 | PON3 | 2349 | TUBB3 |
| 457 | CPNE1 | 1088 | LARP1 | 1719 | POPDC3 | 2350 | TUBBP5 |
| 458 | CPNE8 | 1089 | LARS | 1720 | POR | 2351 | TUBG2 |
| 459 | CPSF6 | 1090 | LARS2 | 1721 | POT1 | 2352 | TULP3 |
| 460 | CRABP2 | 1091 | LBP | 1722 | PP7080 | 2353 | TUSC3 |
| 461 | CRAT | 1092 | LCA5L | 1723 | PPARGC1A | 2354 | TXNDC15 |
| 462 | CREB3L2 | 1093 | LCAL1 | 1724 | PPFIBP1 | 2355 | TYW1 |
| 463 | CRIP2 | 1094 | LCMT1 | 1725 | PPIC | 2356 | TYW1B |
| 464 | CRNDE | 1095 | LDLRAP1 | 1726 | PPL | 2357 | UBA1 |
| 465 | CRNKL1 | 1096 | LDOC1 | 1727 | PPM1H | 2358 | UBA6-AS1 |
| 466 | CRYBB2P1 | 1097 | LEMD2 | 1728 | PPM1L | 2359 | UBE2H |
| 467 | CRYBG3 | 1098 | LEMD3 | 1729 | PPP1R36 | 2360 | UBE2Q2P1 |
| 468 | CSF1 | 1099 | LEPR | 1730 | PPP2CB | 2361 | UBE2Z |
| 469 | CSNK1D | 1100 | LEPROT | 1731 | PRDX2 | 2362 | UBE4A |
| 470 | CST3 | 1101 | LGALS3BP | 1732 | PRDX4 | 2363 | UBFD1 |
| 471 | CST4 | 1102 | LGR4 | 1733 | PRELID2 | 2364 | UBLCP1 |
| 472 | CST6 | 1103 | LGR6 | 1734 | PRELID3B | 2365 | UBN1 |
| 473 | CSTF3 | 1104 | LHX4-AS1 | 1735 | PRICKLE2-AS3 | 2366 | UBR5 |
| 474 | CTAGE5 | 1105 | LIFR | 1736 | PRKAB1 | 2367 | UBXN10 |
| 475 | CTDSP2 | 1106 | LIFR-AS1 | 1737 | PRKAR2B | 2368 | UCHL1 |
| 476 | CTF1 | 1107 | LIMA1 | 1738 | PRKCA | 2369 | UCKL1 |
| 477 | CTNNA1 | 1108 | LIN7C | 1739 | PRKD1 | 2370 | UFL1 |
| 478 | CTSD | 1109 | LINC00174 | 1740 | PRKY | 2371 | UFSP1 |
| 479 | CTSE | 1110 | LINC00476 | 1741 | PRLR | 2372 | UGDH-AS1 |
| 480 | CTSF | 1111 | LINC00482 | 1742 | PRODH | 2373 | UGT2B7 |
| 481 | CTSO | 1112 | LINC00493 | 1743 | PROM2 | 2374 | UGT8 |
| 482 | CTTNBP2 | 1113 | LINC00504 | 1744 | PRORSD1P | 2375 | UHRF1BP1 |
| 483 | CUL5 | 1114 | LINC00506 | 1745 | PRPF8 | 2376 | ULBP2 |
| 484 | CUL7 | 1115 | LINC00578 | 1746 | PRR15L | 2377 | ULBP3 |
| 485 | CWC27 | 1116 | LINC00632 | 1747 | PRR16 | 2378 | UNC5CL |
| 486 | CX3CL1 | 1117 | LINC00673 | 1748 | PRRC1 | 2379 | UPF1 |
| 487 | CXCL12 | 1118 | LINC00674 | 1749 | PRRG2 | 2380 | UQCC1 |
| 488 | CXCL14 | 1119 | LINC00680 | 1750 | PRRG4 | 2381 | UQCRQ |
| 489 | CXCL17 | 1120 | LINC00863 | 1751 | PRSS1 | 2382 | URB1-AS1 |
| 490 | CXCL6 | 1121 | LINC00865 | 1752 | PRSS23 | 2383 | USE1 |
| 491 | CXorf23 | 1122 | LINC00888 | 1753 | PRSS3 | 2384 | USP11 |
| 492 | CXorf56 | 1123 | LINC00909 | 1754 | PRSS3P2 | 2385 | USP15 |
| 493 | CXXC5 | 1124 | LINC00920 | 1755 | PRSS8 | 2386 | USP30 |
| 494 | CYB561 | 1125 | LINC00959 | 1756 | PRUNE1 | 2387 | USP32P2 |
| 495 | CYB561D1 | 1126 | LINC00997 | 1757 | PSD3 | 2388 | USP46 |
| 496 | CYB5A | 1127 | LINC01000 | 1758 | PSENEN | 2389 | USP9X |
| 497 | CYB5R1 | 1128 | LINC01004 | 1759 | PSKH1 | 2390 | UTP11 |
| 498 | CYB5R3 | 1129 | LINC01021 | 1760 | PSMA2 | 2391 | UTP20 |
| 499 | CYHR1 | 1130 | LINC01023 | 1761 | PSMA6 | 2392 | UXT |
| 500 | CYP24A1 | 1131 | LINC01061 | 1762 | PSMB10 | 2393 | VAC14 |
| 501 | CYP27B1 | 1132 | LINC01138 | 1763 | PSMB5 | 2394 | VAC14-AS1 |
| 502 | CYP27C1 | 1133 | LINC01207 | 1764 | PSMC2 | 2395 | VARS |
| 503 | CYP2B7P | 1134 | LINC01214 | 1765 | PSMD7 | 2396 | VASN |
| 504 | CYP4X1 | 1135 | LINC01252 | 1766 | PSMF1 | 2397 | VCAN |
| 505 | CYP51A1 | 1136 | LINC01278 | 1767 | PTCH1 | 2398 | VCL |
| 506 | CYP7B1 | 1137 | LINC01338 | 1768 | PTGER2 | 2399 | VEGFA |
| 507 | CYTH3 | 1138 | LINC01460 | 1769 | PTGES | 2400 | VIPR1 |
| 508 | DAB2IP | 1139 | LINC01465 | 1770 | PTGFRN | 2401 | VKORC1 |
| 509 | DAD1 | 1140 | LINC01479 | 1771 | PTK2 | 2402 | VMP1 |
| 510 | DAG1 | 1141 | LINC01481 | 1772 | PTOV1 | 2403 | VOPP1 |
| 511 | DANCR | 1142 | LINC01521 | 1773 | PTOV1-AS1 | 2404 | VPS16 |
| 512 | DANT2 | 1143 | LINC01578 | 1774 | PTOV1-AS2 | 2405 | VPS18 |
| 513 | DAP | 1144 | LIPK | 1775 | PTPA | 2406 | VPS25 |
| 514 | DBN1 | 1145 | LIPT1 | 1776 | PTPRF | 2407 | VPS4A |
| 515 | DCAF13 | 1146 | LLGL2 | 1777 | PTPRU | 2408 | VPS50 |
| 516 | DCAF4 | 1147 | LLPH | 1778 | PTRH2 | 2409 | VPS9D1 |
| 517 | DCBLD2 | 1148 | LMAN1 | 1779 | PTTG1IP | 2410 | VSTM2L |
| 518 | DCHS1 | 1149 | LMAN2 | 1780 | PUDP | 2411 | VSTM4 |
| 519 | DCLK1 | 1150 | LMAN2L | 1781 | PUM3 | 2412 | VSX1 |
| 520 | DCP1B | 1151 | LMO3 | 1782 | PURA | 2413 | VTRNA1-2 |
| 521 | DCTN2 | 1152 | LNPK | 1783 | PWARSN | 2414 | VTRNA1-3 |
| 522 | DCUN1D3 | 1153 | LOC100126784 | 1784 | PWWP2B | 2415 | VWA1 |
| 523 | DCUN1D4 | 1154 | LOC100129034 | 1785 | PXDN | 2416 | VWA2 |
| 524 | DDAH1 | 1155 | LOC100129434 | 1786 | PXMP4 | 2417 | VWDE |
| 525 | DDIT3 | 1156 | LOC100129931 | 1787 | PXN | 2418 | WAC-AS1 |
| 526 | DDIT4 | 1157 | LOC100129940 | 1788 | PYCR1 | 2419 | WASF1 |
| 527 | DDIT4L | 1158 | LOC100130111 | 1789 | PYGB | 2420 | WBP1L |
| 528 | DDR1 | 1159 | LOC100130691 | 1790 | QPCT | 2421 | WDFY3 |
| 529 | DDX41 | 1160 | LOC100131257 | 1791 | R3HDM2 | 2422 | WDR13 |
| 530 | DDX46 | 1161 | LOC100131289 | 1792 | RAB10 | 2423 | WDR19 |
| 531 | DDX47 | 1162 | LOC100132249 | 1793 | RAB24 | 2424 | WDR24 |
| 532 | DDX60 | 1163 | LOC100132356 | 1794 | RAB27A | 2425 | WDR36 |
| 533 | DDX60L | 1164 | LOC100133091 | 1795 | RAB27B | 2426 | WDR45 |
| 534 | DECR2 | 1165 | LOC100134868 | 1796 | RAB36 | 2427 | WDR59 |
| 535 | DEFB4A | 1166 | LOC100270746 | 1797 | RAB3IP | 2428 | WDR60 |
| 536 | DENND2A | 1167 | LOC100270804 | 1798 | RAB40C | 2429 | WFDC10B |
| 537 | DENND4C | 1168 | LOC100272217 | 1799 | RABAC1 | 2430 | WFDC2 |
| 538 | DEXI | 1169 | LOC100287015 | 1800 | RABGEF1 | 2431 | WFDC3 |
| 539 | DFNA5 | 1170 | LOC100287896 | 1801 | RACK1 | 2432 | WFIKKN1 |
| 540 | DGCR2 | 1171 | LOC100288069 | 1802 | RAD50 | 2433 | WHAMMP1 |
| 541 | DGCR6 | 1172 | LOC100288152 | 1803 | RAD51B | 2434 | WNK3 |
| 542 | DGKQ | 1173 | LOC100288748 | 1804 | RAI2 | 2435 | WNT5B |
| 543 | DHRS3 | 1174 | LOC100288846 | 1805 | RALGAPA2 | 2436 | WRB |
| 544 | DHRS4-AS1 | 1175 | LOC100289230 | 1806 | RALGPS2 | 2437 | WWC1 |
| 545 | DHX29 | 1176 | LOC100419583 | 1807 | RALY-AS1 | 2438 | XDH |
| 546 | DHX35 | 1177 | LOC100505771 | 1808 | RAMP1 | 2439 | XKRX |
| 547 | DIABLO | 1178 | LOC100505938 | 1809 | RANBP17 | 2440 | XPOT |
| 548 | DICER1-AS1 | 1179 | LOC100505984 | 1810 | RAP1B | 2441 | YEATS4 |
| 549 | DIO1 | 1180 | LOC100506083 | 1811 | RAPH1 | 2442 | YIF1A |
| 550 | DKFZP434I0714 | 1181 | LOC100506098 | 1812 | RARRES3 | 2443 | YIPF5 |
| 551 | DKK3 | 1182 | LOC100506127 | 1813 | RARS | 2444 | YIPF6 |
| 552 | DLG3 | 1183 | LOC100506178 | 1814 | RASAL2 | 2445 | YKT6 |
| 553 | DLGAP4 | 1184 | LOC100506314 | 1815 | RASD1 | 2446 | YTHDC2 |
| 554 | DNAH11 | 1185 | LOC100506548 | 1816 | RASD2 | 2447 | ZAK |
| 555 | DNAH2 | 1186 | LOC100506603 | 1817 | RASEF | 2448 | ZBED8 |
| 556 | DNAH6 | 1187 | LOC100506746 | 1818 | RASGRF2 | 2449 | ZBED9 |
| 557 | DNAH7 | 1188 | LOC100506844 | 1819 | RASSF3 | 2450 | ZBTB20 |
| 558 | DNAJA3 | 1189 | LOC100506990 | 1820 | RASSF6 | 2451 | ZBTB39 |
| 559 | DNAJB12 | 1190 | LOC100507053 | 1821 | RBCK1 | 2452 | ZC2HC1A |
| 560 | DNAJB14 | 1191 | LOC100507291 | 1822 | RBFOX2 | 2453 | ZDHHC16 |
| 561 | DNAJC12 | 1192 | LOC100507577 | 1823 | RBKS | 2454 | ZDHHC6 |
| 562 | DNAJC15 | 1193 | LOC100507642 | 1824 | RBM12 | 2455 | ZDHHC7 |
| 563 | DNAJC17 | 1194 | LOC100652758 | 1825 | RBM41 | 2456 | ZDHHC9 |
| 564 | DNAJC9-AS1 | 1195 | LOC100996255 | 1826 | RBMS3 | 2457 | ZER1 |
| 565 | DNAL1 | 1196 | LOC100996419 | 1827 | RBPMS | 2458 | ZFP62 |
| 566 | DNALI1 | 1197 | LOC100996842 | 1828 | RBSN | 2459 | ZFP90 |
| 567 | DNER | 1198 | LOC101926898 | 1829 | RCBTB1 | 2460 | ZFPM2-AS1 |
| 568 | DNMBP | 1199 | LOC101926935 | 1830 | RCC1L | 2461 | ZFR |
| 569 | DNTTIP1 | 1200 | LOC101927168 | 1831 | RDH16 | 2462 | ZFR2 |
| 570 | DOCK1 | 1201 | LOC101927391 | 1832 | REEP5 | 2463 | ZFYVE16 |
| 571 | DOCK3 | 1202 | LOC101927418 | 1833 | REEP6 | 2464 | ZKSCAN4 |
| 572 | DOCK9-AS2 | 1203 | LOC101927420 | 1834 | REG1A | 2465 | ZMAT1 |
| 573 | DOK5 | 1204 | LOC101927630 | 1835 | REM2 | 2466 | ZMAT3 |
| 574 | DPH6 | 1205 | LOC101927653 | 1836 | RERE | 2467 | ZNF133 |
| 575 | DPP4 | 1206 | LOC101927755 | 1837 | RERG | 2468 | ZNF134 |
| 576 | DPY19L1 | 1207 | LOC101927765 | 1838 | REXO2 | 2469 | ZNF185 |
| 577 | DPY19L1P1 | 1208 | LOC101927780 | 1839 | RFNG | 2470 | ZNF2 |
| 578 | DPY19L2 | 1209 | LOC101927809 | 1840 | RGMB | 2471 | ZNF202 |
| 579 | DPY19L3 | 1210 | LOC101927811 | 1841 | RGS14 | 2472 | ZNF204P |
| 580 | DPYD | 1211 | LOC101927972 | 1842 | RGS5 | 2473 | ZNF213 |
| 581 | DPYSL3 | 1212 | LOC101928069 | 1843 | RHBDF1 | 2474 | ZNF23 |
| 582 | DSC2 | 1213 | LOC101928093 | 1844 | RHBDL2 | 2475 | ZNF239 |
| 583 | DSG2 | 1214 | LOC101928103 | 1845 | RHOBTB3 | 2476 | ZNF271P |
| 584 | DSG2-AS1 | 1215 | LOC101928111 | 1846 | RHOC | 2477 | ZNF302 |
| 585 | DSP | 1216 | LOC101928295 | 1847 | RHOV | 2478 | ZNF334 |
| 586 | DST | 1217 | LOC101928595 | 1848 | RHPN2 | 2479 | ZNF337 |
| 587 | DSTN | 1218 | LOC101928659 | 1849 | RIC1 | 2480 | ZNF341 |
| 588 | DSTNP2 | 1219 | LOC101928794 | 1850 | RIC3 | 2481 | ZNF347 |
| 589 | DTWD2 | 1220 | LOC101928936 | 1851 | RIDA | 2482 | ZNF354A |
| 590 | DTX2 | 1221 | LOC101929125 | 1852 | RIN1 | 2483 | ZNF354B |
| 591 | DTX3 | 1222 | LOC101929147 | 1853 | RIN2 | 2484 | ZNF395 |
| 592 | DUS4L | 1223 | LOC101929161 | 1854 | RIOK2 | 2485 | ZNF415 |
| 593 | DUSP1 | 1224 | LOC101929188 | 1855 | RLN2 | 2486 | ZNF429 |
| 594 | DUSP23 | 1225 | LOC101929567 | 1856 | RMND5B | 2487 | ZNF43 |
| 595 | DUSP6 | 1226 | LOC101929705 | 1857 | RNASE1 | 2488 | ZNF446 |
| 596 | DYNC1H1 | 1227 | LOC101929709 | 1858 | RNASE4 | 2489 | ZNF454 |
| 597 | DYNC1LI2 | 1228 | LOC101929767 | 1859 | RNASEL | 2490 | ZNF490 |
| 598 | DYNC2H1 | 1229 | LOC101930071 | 1860 | RNF10 | 2491 | ZNF507 |
| 599 | DYNC2LI1 | 1230 | LOC101930085 | 1861 | RNF128 | 2492 | ZNF561-AS1 |
| 600 | DYRK2 | 1231 | LOC102467147 | 1862 | RNF14 | 2493 | ZNF595 |
| 601 | DYSF | 1232 | LOC102723345 | 1863 | RNF149 | 2494 | ZNF605 |
| 602 | DZANK1 | 1233 | LOC102723354 | 1864 | RNF175 | 2495 | ZNF621 |
| 603 | DZIP1 | 1234 | LOC102724094 | 1865 | RNF212 | 2496 | ZNF622 |
| 604 | EBLN3P | 1235 | LOC102724156 | 1866 | RNF216P1 | 2497 | ZNF646 |
| 605 | ECE1 | 1236 | LOC102724312 | 1867 | RNF26 | 2498 | ZNF652 |
| 606 | EDA | 1237 | LOC102724699 | 1868 | RNF40 | 2499 | ZNF668 |
| 607 | EDA2R | 1238 | LOC102724784 | 1869 | RNLS | 2500 | ZNF680 |
| 608 | EDEM2 | 1239 | LOC102724927 | 1870 | RNPC3 | 2501 | ZNF688 |
| 609 | EDIL3 | 1240 | LOC103611081 | 1871 | RNR1 | 2502 | ZNF710 |
| 610 | EDN2 | 1241 | LOC105369340 | 1872 | ROGDI | 2503 | ZNF711 |
| 611 | EDNRA | 1242 | LOC105369632 | 1873 | ROMO1 | 2504 | ZNF718 |
| 612 | EDRF1 | 1243 | LOC105369635 | 1874 | ROS1 | 2505 | ZNF737 |
| 613 | EEF1AKMT1 | 1244 | LOC105370333 | 1875 | RPARP-AS1 | 2506 | ZNF746 |
| 614 | EEF1G | 1245 | LOC105371743 | 1876 | RPGRIP1L | 2507 | ZNF747 |
| 615 | EEF2K | 1246 | LOC105372582 | 1877 | RPL26L1 | 2508 | ZNF75A |
| 616 | EFCAB13 | 1247 | LOC105373383 | 1878 | RPL32P3 | 2509 | ZNF767P |
| 617 | EFEMP1 | 1248 | LOC105374366 | 1879 | RPN2 | 2510 | ZNF789 |
| 618 | EFL1 | 1249 | LOC105376805 | 1880 | RPP14 | 2511 | ZNF805 |
| 619 | EFNA4 | 1250 | LOC105377458 | 1881 | RPS14P3 | 2512 | ZNF814 |
| 620 | EFNA5 | 1251 | LOC105377621 | 1882 | RPSAP9 | 2513 | ZNF827 |
| 621 | EGFR | 1252 | LOC105377763 | 1883 | RRAGA | 2514 | ZNF839 |
| 622 | EGLN1 | 1253 | LOC105378853 | 1884 | RRN3 | 2515 | ZNF853 |
| 623 | EGLN3 | 1254 | LOC107133515 | 1885 | RSPRY1 | 2516 | ZNF862 |
| 624 | EHD2 | 1255 | LOC146880 | 1886 | RSRP1 | 2517 | ZNHIT1 |
| 625 | EHF | 1256 | LOC154761 | 1887 | RTKN | 2518 | ZNRD1ASP |
| 626 | EIF2AK4 | 1257 | LOC202181 | 1888 | RTN4IP1 | 2519 | ZSCAN12 |
| 627 | EIF4G3 | 1258 | LOC257396 | 1889 | RTN4RL1 | 2520 | ZSCAN30 |
| 628 | ELL3 | 1259 | LOC283710 | 1890 | RTN4RL2 | 2521 | ZSWIM1 |
| 629 | ELMO2 | 1260 | LOC284023 | 1891 | RTP4 |  |  |
| 630 | ELMO3 | 1261 | LOC284578 | 1892 | RUNDC3B |  |  |
| 631 | ELOVL6 | 1262 | LOC284798 | 1893 | RWDD2B |  |  |
