# Supplementary material for: Leveraging Genetic Instrumental Variables and Sequencing Analysis to Identify a Prognostic Signature Based on Epithelial Cell Markers in Lung Adenocarcinoma
Source: Thorac Cancer. 2026 Jan 7;17(1):e70244. doi: 10.1111/1759-7714.70244 (PMC12779403; doi:10.1111/1759-7714.70244)
Supplement: Supplementary file 4 — Table S3: Genes from the IEU database with p_SMR values greater than 0.01 and HEIDI values less than 0.01 (n = 226). [file TCA-17-e70244-s001.docx]

Supplementary Table 3 Genes from the IEU database with p-SMR values greater than 0.01 and HEIDI values less than 0.01.(n=226)

| Gene | b_SMR | se_SMR | p_SMR | p_HEIDI |
| --- | --- | --- | --- | --- |
| REXO5 | 0.592 | 0.220 | 0.007 | 0.836 |
| MARK4 | -0.137 | 0.045 | 0.002 | 0.498 |
| NFIX | 0.689 | 0.252 | 0.006 | 0.608 |
| APBA3 | -0.301 | 0.112 | 0.007 | 0.065 |
| ERCC1 | -0.191 | 0.061 | 0.002 | 0.125 |
| MNAT1 | 0.555 | 0.198 | 0.005 | 0.340 |
| GRAMD1B | 0.315 | 0.120 | 0.009 | 0.517 |
| TYMP | -0.090 | 0.027 | <0.001 | 0.253 |
| RNASET2 | 0.070 | 0.018 | <0.001 | 0.812 |
| RIPOR1 | 0.234 | 0.091 | 0.010 | 0.369 |
| LIMA1 | 0.147 | 0.049 | 0.003 | 0.401 |
| RC3H2 | -0.405 | 0.141 | 0.004 | 0.592 |
| RNF4 | 0.373 | 0.141 | 0.008 | 0.547 |
| CASP8 | -0.217 | 0.079 | 0.006 | 0.111 |
| ST3GAL6 | 0.174 | 0.064 | 0.006 | 0.451 |
| RFXANK | 0.175 | 0.055 | 0.002 | 0.480 |
| ANKRD44 | 0.320 | 0.123 | 0.009 | 0.505 |
| POLR1H | -0.119 | 0.043 | 0.006 | 0.575 |
| STAG3 | -0.105 | 0.039 | 0.007 | 0.418 |
| RPS6KA2 | 0.197 | 0.052 | <0.001 | 0.153 |
| MCM2 | 0.632 | 0.204 | 0.002 | 0.636 |
| NUP37 | -0.650 | 0.217 | 0.003 | 0.061 |
| GRAMD4 | -0.124 | 0.036 | <0.001 | 0.089 |
| ICAM3 | -0.130 | 0.050 | 0.009 | 0.504 |
| XAB2 | 0.194 | 0.070 | 0.006 | 0.158 |
| STXBP2 | -0.223 | 0.074 | 0.003 | 0.059 |
| SDF4 | -0.180 | 0.066 | 0.007 | 0.906 |
| SLC27A5 | 0.538 | 0.199 | 0.007 | 0.877 |
| DIMT1 | 0.269 | 0.096 | 0.005 | 0.090 |
| SNX5 | 0.320 | 0.114 | 0.005 | 0.334 |
| GCN1 | -0.121 | 0.044 | 0.006 | 0.522 |
| PSMD5 | -0.252 | 0.079 | 0.001 | 0.418 |
| POLRMT | 0.453 | 0.125 | <0.001 | 0.343 |
| MKNK2 | 0.166 | 0.063 | 0.009 | 0.227 |
| BCL2L13 | 0.096 | 0.034 | 0.005 | 0.481 |
| FOXRED2 | -0.105 | 0.034 | 0.002 | 0.505 |
| PVALB | -0.081 | 0.028 | 0.004 | 0.864 |
| PSMA6 | -0.384 | 0.149 | 0.010 | 0.557 |
| NFKBIA | -0.159 | 0.051 | 0.002 | 0.947 |
| CD40 | -0.111 | 0.036 | 0.002 | 0.582 |
| ACTR5 | 0.117 | 0.037 | 0.002 | 0.218 |
| CEP192 | -0.069 | 0.020 | <0.001 | 0.844 |
| POLI | -0.095 | 0.033 | 0.003 | 0.391 |
| FLT1 | -0.172 | 0.062 | 0.006 | 0.133 |
| ACD | 0.304 | 0.114 | 0.008 | 0.458 |
| NECAB2 | 0.144 | 0.043 | <0.001 | 0.830 |
| HSDL1 | 0.154 | 0.054 | 0.004 | 0.854 |
| TAF1C | 0.087 | 0.030 | 0.004 | 0.848 |
| ATP8B4 | 0.113 | 0.037 | 0.002 | 0.080 |
| GYS1 | 0.326 | 0.121 | 0.007 | 0.486 |
| ICAM5 | -0.394 | 0.122 | 0.001 | 0.329 |
| TYK2 | 0.196 | 0.073 | 0.007 | 0.342 |
| MYH3 | 0.163 | 0.063 | 0.010 | 0.346 |
| CD5 | -0.215 | 0.083 | 0.010 | 0.189 |
| KHDRBS2 | -0.435 | 0.167 | 0.009 | 0.209 |
| TRIM38 | -0.168 | 0.063 | 0.007 | 0.318 |
| BTNL8 | 0.081 | 0.030 | 0.007 | 0.089 |
| ZAP70 | -0.203 | 0.073 | 0.006 | 0.783 |
| EHBP1 | -0.954 | 0.306 | 0.002 | 0.889 |
| SRSF4 | 0.530 | 0.192 | 0.006 | 0.215 |
| GNPAT | -0.240 | 0.085 | 0.005 | 0.719 |
| SIPA1L2 | 0.127 | 0.043 | 0.003 | 0.740 |
| RPS6KA1 | -0.243 | 0.082 | 0.003 | 0.540 |
| DHDDS | 0.497 | 0.154 | 0.001 | 0.359 |
| NSL1 | -0.204 | 0.063 | 0.001 | 0.334 |
| DDX59 | -0.084 | 0.027 | 0.002 | 0.190 |
| MYL12B | -0.574 | 0.166 | <0.001 | 0.505 |
| CTNNAL1 | -0.125 | 0.046 | 0.006 | 0.841 |
| FBXW2 | 0.469 | 0.156 | 0.003 | 0.331 |
| NEK6 | -0.089 | 0.032 | 0.006 | 0.732 |
| CFAP58 | 0.362 | 0.121 | 0.003 | 0.347 |
| TEK | 0.103 | 0.036 | 0.005 | 0.280 |
| ARL1 | 0.264 | 0.100 | 0.008 | 0.662 |
| SEC22A | 0.340 | 0.103 | <0.001 | 0.527 |
| ACVR2A | -0.213 | 0.074 | 0.004 | 0.746 |
| PTGFR | -0.410 | 0.109 | <0.001 | 0.313 |
| CCDC91 | 0.289 | 0.106 | 0.006 | 0.650 |
| ATF1 | -0.173 | 0.053 | 0.001 | 0.156 |
| F13A1 | 0.134 | 0.050 | 0.008 | 0.347 |
| H2BC11 | -0.268 | 0.091 | 0.003 | 0.065 |
| HROB | -0.355 | 0.134 | 0.008 | 0.998 |
| FNDC11 | -0.834 | 0.269 | 0.002 | 0.213 |
| MGME1 | 0.077 | 0.027 | 0.005 | 0.272 |
| KLC1 | 0.121 | 0.046 | 0.009 | 0.555 |
| IRF3 | 0.181 | 0.064 | 0.004 | 0.170 |
| GMFG | 0.353 | 0.092 | <0.001 | 0.122 |
| KCNC3 | 0.488 | 0.181 | 0.007 | 0.653 |
| NAPSA | -0.215 | 0.078 | 0.006 | 0.471 |
| NAPSB | -0.058 | 0.020 | 0.003 | 0.874 |
| SCO1 | -0.102 | 0.039 | 0.009 | 0.320 |
| MYH11 | -0.091 | 0.033 | 0.006 | 0.065 |
| MPHOSPH6 | 0.102 | 0.028 | <0.001 | 0.484 |
| MFSD9 | -0.069 | 0.025 | 0.006 | 0.239 |
| TBC1D4 | 0.113 | 0.041 | 0.005 | 0.679 |
| FLOT1 | 0.185 | 0.047 | <0.001 | 0.112 |
| IER3 | 0.187 | 0.047 | <0.001 | 0.059 |
| VARS2 | -0.242 | 0.068 | <0.001 | 0.066 |
| TGS1 | -0.167 | 0.048 | <0.001 | 0.430 |
| SECISBP2L | -0.898 | 0.210 | <0.001 | 0.147 |
| INTS12 | 0.382 | 0.127 | 0.003 | 0.683 |
| HADH | 0.359 | 0.112 | 0.001 | 0.061 |
| GRTP1 | -0.332 | 0.113 | 0.003 | 0.093 |
| PELI2 | 0.250 | 0.092 | 0.007 | 0.385 |
| DISP2 | 0.253 | 0.089 | 0.004 | 0.134 |
| MCTP2 | -0.208 | 0.079 | 0.008 | 0.289 |
| MBTPS1 | 0.114 | 0.036 | 0.002 | 0.682 |
| MINK1 | 0.136 | 0.049 | 0.006 | 0.129 |
| DYM | -0.224 | 0.075 | 0.003 | 0.128 |
| NTN5 | 0.333 | 0.072 | <0.001 | 0.619 |
| NOSIP | 0.276 | 0.098 | 0.005 | 0.187 |
| GPR161 | 0.499 | 0.188 | 0.008 | 0.287 |
| NAF1 | -0.935 | 0.215 | <0.001 | 0.466 |
| COMMD10 | 0.150 | 0.052 | 0.004 | 0.850 |
| MMS22L | -0.398 | 0.123 | 0.001 | 0.110 |
| INIP | 0.188 | 0.064 | 0.003 | 0.115 |
| LRSAM1 | 0.378 | 0.145 | 0.009 | 0.094 |
| GSTO1 | 0.262 | 0.087 | 0.002 | 0.266 |
| ADM | -0.129 | 0.049 | 0.008 | 0.655 |
| CCDC81 | -0.581 | 0.198 | 0.003 | 0.465 |
| MPZL2 | -0.152 | 0.024 | <0.001 | 0.136 |
| SAP18 | -0.414 | 0.145 | 0.004 | 0.685 |
| IL18 | 0.193 | 0.061 | 0.002 | 0.740 |
| TMEM86A | 0.362 | 0.133 | 0.007 | 0.847 |
| WWC2 | -0.470 | 0.170 | 0.006 | 0.225 |
| FBXL18 | 0.643 | 0.239 | 0.007 | 0.083 |
| KLF10 | -0.393 | 0.145 | 0.007 | 0.470 |
| ZDHHC5 | 0.191 | 0.069 | 0.005 | 0.857 |
| WASF2 | -0.591 | 0.222 | 0.008 | 0.798 |
| RAPGEF6 | -0.310 | 0.102 | 0.002 | 0.497 |
| MED27 | 0.562 | 0.159 | <0.001 | 0.653 |
| MPZL3 | -0.404 | 0.064 | <0.001 | 0.224 |
| GPATCH4 | 0.470 | 0.177 | 0.008 | 0.842 |
| DMKN | -0.576 | 0.159 | <0.001 | 0.356 |
| FGF11 | 0.919 | 0.260 | <0.001 | 0.538 |
| DNAI3 | -0.158 | 0.053 | 0.003 | 0.245 |
| DISC1 | -0.134 | 0.043 | 0.002 | 0.391 |
| HPS3 | 0.259 | 0.086 | 0.003 | 0.211 |
| SNIP1 | 0.199 | 0.069 | 0.004 | 0.440 |
| CFAP100 | 0.956 | 0.294 | 0.001 | 0.471 |
| SMIM43 | 0.827 | 0.294 | 0.005 | 0.330 |
| METTL27 | -0.051 | 0.019 | 0.008 | 0.351 |
| AQP3 | 0.321 | 0.097 | <0.001 | 0.167 |
| TTC7B | 0.620 | 0.220 | 0.005 | 0.399 |
| CNPY4 | -0.167 | 0.060 | 0.005 | 0.101 |
| CORO6 | -0.353 | 0.127 | 0.005 | 0.213 |
| CYP2S1 | -0.110 | 0.038 | 0.004 | 0.769 |
| TNXB | -0.115 | 0.039 | 0.003 | 0.264 |
| BTNL3 | -0.058 | 0.021 | 0.007 | 0.060 |
| KLF13 | 0.483 | 0.159 | 0.002 | 0.456 |
| ADPRM | -0.261 | 0.096 | 0.007 | 0.060 |
| CHCHD7 | -0.299 | 0.115 | 0.010 | 0.715 |
| CTPS1 | -0.140 | 0.049 | 0.004 | 0.375 |
| SNTB1 | 0.429 | 0.145 | 0.003 | 0.068 |
| CLP1 | 0.437 | 0.163 | 0.007 | 0.751 |
| FAM241A | -0.137 | 0.047 | 0.004 | 0.228 |
| PCP2 | 0.145 | 0.040 | <0.001 | 0.338 |
| CD164L2 | -0.248 | 0.087 | 0.004 | 0.842 |
| ABO | 0.076 | 0.028 | 0.007 | 0.466 |
| TUBB6 | 0.073 | 0.027 | 0.007 | 0.449 |
| CDC26 | -0.118 | 0.034 | <0.001 | 0.528 |
| B3GNT5 | 0.253 | 0.077 | 0.001 | 0.811 |
| NINJ2-AS1 | 0.073 | 0.024 | 0.002 | 0.367 |
| LINC00324 | 0.077 | 0.028 | 0.006 | 0.638 |
| SAMD4B | -0.350 | 0.121 | 0.004 | 0.051 |
| FUCA1 | 0.094 | 0.036 | 0.010 | 0.649 |
| LOC100128310 | 0.151 | 0.057 | 0.008 | 0.269 |
| ARID3B | -0.367 | 0.099 | <0.001 | 0.393 |
| COA4 | -0.688 | 0.225 | 0.002 | 0.434 |
| CAMK1D | -0.104 | 0.031 | <0.001 | 0.087 |
| RUVBL2 | -0.219 | 0.081 | 0.007 | 0.384 |
| MRGPRE | 0.300 | 0.096 | 0.002 | 0.646 |
| TRARG1 | -0.441 | 0.152 | 0.004 | 0.212 |
| ZNF17 | 0.604 | 0.228 | 0.008 | 0.622 |
| TMEM220 | -0.196 | 0.069 | 0.005 | 0.770 |
| RINL | -0.248 | 0.090 | 0.006 | 0.346 |
| TMPPE | 1.084 | 0.360 | 0.003 | 0.109 |
| NUTM2B | -0.107 | 0.029 | <0.001 | 0.648 |
| HYKK | -4.155 | 0.762 | <0.001 | 0.460 |
| SELL | 0.083 | 0.028 | 0.003 | 0.957 |
| ACTMAP | -0.134 | 0.049 | 0.006 | 0.112 |
| CNR2 | -0.468 | 0.178 | 0.009 | 0.101 |
| ZNF138 | -0.158 | 0.054 | 0.003 | 0.622 |
| ZNF257 | 0.228 | 0.086 | 0.008 | 0.107 |
| GZMM | -0.302 | 0.100 | 0.002 | 0.360 |
| PARVA | -0.455 | 0.127 | <0.001 | 0.063 |
| UCKL1 | -0.231 | 0.060 | <0.001 | 0.207 |
| FAM3D | -0.325 | 0.100 | 0.001 | 0.079 |
| ZNF358 | -0.404 | 0.147 | 0.006 | 0.135 |
| HLA-DMA | 0.235 | 0.085 | 0.006 | 0.307 |
| NOTCH4 | 0.396 | 0.137 | 0.004 | 0.324 |
| PRRT1 | 0.773 | 0.221 | <0.001 | 0.435 |
| LOC100507547 | 0.773 | 0.221 | <0.001 | 0.435 |
| APOM | -0.511 | 0.151 | <0.001 | 0.151 |
| CCHCR1 | 0.143 | 0.054 | 0.008 | 0.071 |
| HLA-G | -0.097 | 0.031 | 0.002 | 0.102 |
| LINC02210 | -0.073 | 0.022 | <0.001 | 0.863 |
| TMX2 | 0.148 | 0.055 | 0.007 | 0.757 |
| KANSL1-AS1 | -0.060 | 0.017 | <0.001 | 0.411 |
| LINC00243 | 0.125 | 0.031 | <0.001 | 0.505 |
| CRPPA | -0.247 | 0.088 | 0.005 | 0.459 |
| RTL10 | -0.121 | 0.041 | 0.003 | 0.311 |
| NBPF1 | 0.157 | 0.060 | 0.009 | 0.274 |
| PPT2 | 0.356 | 0.106 | <0.001 | 0.066 |
| IGLC6 | 0.096 | 0.036 | 0.008 | 0.352 |
| C4B | 0.087 | 0.024 | <0.001 | 0.193 |
| AP4B1-AS1 | -0.190 | 0.073 | 0.009 | 0.490 |
| CUTALP | -0.052 | 0.016 | 0.001 | 0.248 |
| NUTM2E | 0.122 | 0.036 | <0.001 | 0.523 |
| FBXW4P1 | 0.508 | 0.143 | <0.001 | 0.127 |
| HLA-DPA1 | 0.066 | 0.025 | 0.009 | 0.369 |
| CYP21A2 | 0.221 | 0.060 | <0.001 | 0.134 |
| TRIM26 | 0.296 | 0.097 | 0.002 | 0.253 |
| LOC613206 | -0.217 | 0.082 | 0.008 | 0.914 |
| LRRC37A2 | -0.071 | 0.021 | <0.001 | 0.447 |
| C4A | -0.175 | 0.048 | <0.001 | 0.160 |
| LOC110384692 | -0.175 | 0.048 | <0.001 | 0.160 |
| PDLIM1P4 | 0.103 | 0.034 | 0.002 | 0.601 |
| GOLGA8K | -0.302 | 0.113 | 0.007 | 0.096 |
| ENPP7P11 | 0.196 | 0.068 | 0.004 | 0.438 |
| STX16-NPEPL1 | -0.265 | 0.092 | 0.004 | 0.092 |
| POLR2M | -0.211 | 0.080 | 0.008 | 0.397 |
| PRORP | -0.092 | 0.029 | 0.002 | 0.872 |
| AFTPH-DT | -0.140 | 0.050 | 0.005 | 0.418 |
| MAPK8IP1P1 | -0.208 | 0.060 | <0.001 | 0.718 |
| TMEM220-AS1 | -0.238 | 0.092 | 0.010 | 0.324 |
| MIF4GD-DT | 0.149 | 0.056 | 0.008 | 0.132 |
